# INSIGHT Model: Explainable Artificial Intelligence for Real-World EHR-Based Screening of Transthyretin Amyloidosis Cardiomyopathy

**DOI:** 10.1016/j.jacadv.2026.103122

**Published:** 2026-08-26

**Authors:** Akshay Arora, Muhammad Shahzeb Khan, Cynthia Sunderman, Nicole Baldwin, Monica Bennett, Vee Anne Bomar, Lisa Cross, Briget da Graca, Jason Ettlinger, Robert L. Gottlieb, Kendall Hammonds, Michael Mack, Ahmad Masri, Mia Papas, Elisa L. Priest, Courtney Shaver, John Venditto, Javed Butler

**Affiliations:** a Baylor Scott & White Research Institute, Dallas, Texas, USA; b Department of Medicine, Baylor College of Medicine, Temple, Texas, USA; c Baylor Scott & White The Heart Hospital Plano, Plano, Texas, USA; d Baylor Scott & White The Heart Hospital, Dallas, Texas, USA; e Baylor University Medical Center, Dallas, Texas, USA; f Texas A&M Health Science Center, Dallas, Texas, USA; g Burnett School of Medicine at TCU, Fort Worth, Texas, USA; h OHSU-PSU School of Public Health, Oregon Health and Science University, Portland, Oregon, USA; i US Medical Affairs, BioPharmaceutical Medical, AstraZeneca, Wilmington, Delaware, USA; j University of Mississippi Medical Center, Jackson, Mississippi, USA

**Keywords:** artificial intelligence, Clinical ModernBERT, electronic health record data, natural language processing, transthyretin amyloidosis cardiomyopathy

## Abstract

**Background:**

Transthyretin amyloidosis (ATTR) diagnoses are frequently delayed or missed, attenuating treatment benefit.

**Objectives:**

The authors developed an explainable artificial intelligence screening tool to identify high-risk patients who should be considered for diagnostic testing for ATTR-cardiomyopathy (ATTR-CM) from an unselected heart failure (HF) population.

**Methods:**

Electronic health record data (April 2020–March 2023) were used to identify patients with suspected ATTR-CM in an HF cohort. Natural language processing with Named Entity Recognition processed unstructured clinical, echocardiographic, and electrocardiographic data. Clinical ModernBERT predicted suspected ATTR-CM. Local interpretable model-agnostic explanations were provided. The authors examined sensitivity, specificity, positive predictive value, negative predictive value, F1 score, area under the receiver-operating characteristic curve, and key model predictors.

**Results:**

This study included 100,290 patients with HF (mean age: 71.6 ± 14.3 years; 47.3% female; 76.2% White; 18.2% Black; 89.7% non-Hispanic). The Clinical ModernBERT model, INSIGHT-CM, identified suspected ATTR-CM (n = 1,108; prevalence 1.2%) with sensitivity 77.5% (95% CI: 75.9%-82.8%), specificity 94.7% (95% CI: 94.4%-95.0%), positive predictive value 19.9% (95% CI: 17.6%-22.36%), negative predictive value 99.6% (95% CI: 99.5%-99.7%), and area under the receiver-operating characteristic curve 92.4% (95% CI: 90.5%-94.3%).

**Conclusions:**

In an unselected HF population with 1% prevalence of suspected ATTR-CM, INSIGHT-CM identified patients among whom 1 in 5 fit the pattern for suspected ATTR-CM, offering meaningful opportunities to improve timely diagnosis and treatment.

Transthyretin amyloidosis (ATTR) is a fatal infiltrative disease, with an untreated median survival among patients with cardiac ATTR amyloidosis of 3.6 to 4.8 years post-diagnosis.^1^^,^^2^ Almost half of patients die within 24 months of diagnosis.^1^^,^^2^ The true population prevalence of ATTR-cardiomyopathy (ATTR-CM) is unknown but has been estimated at 6.1 per million in the United States,^3^ although this depends on the definition used to identify cases.^4^ Furthermore, precise estimates are difficult to ascertain as many cases are diagnosed late, with delays of 1.3 to 7.2 years from the first documented cardiac symptoms,^5^, ^6^, ^7^ or are missed.^8^^,^^9^ Thus, opportunities for timely intervention to improve outcomes are often missed.^1^^,^^10^

Previous research has sought to identify patients who should be tested for ATTR amyloidosis. The Mayo Clinic Simple Score estimated risk based on clinical variables, showing good discrimination (C-statistic >0.80).^11^ However, when applied to a real-world population of heart failure (HF) patients in which the prevalence of ATTR-CM was <1%, positive predictive value (PPV) was <1%.^12^ Machine learning and deep learning have been used to develop predictive models for ATTR-CM^13^, ^14^, ^15^, ^16^, ^17^, ^18^, ^19^, ^20^, ^21^, ^22^, ^23^ and have shown some promising results, with sensitivity and specificity ranging from 18% to 95% and 66% to 99%.^13^, ^14^, ^15^, ^16^, ^17^, ^18^, ^19^, ^20^, ^21^, ^22^, ^23^ However, in real-world HF populations, these tools face the same PPV challenge related to low ATTR-CM prevalence. For example, in the ALARM (nAtural LanguAge pRocessing in aMyloidosis) study, a model to predict ATTR amyloidosis diagnosis codes was developed by applying natural language processing algorithms to clinical notes in electronic health record (EHR) data from an integrated health care system; when applied to >100,000 patients with a diagnosis of HF and/or neuropathy, PPV for ATTR amyloidosis was only 3.4%, despite a sensitivity of 74% and specificity of 87%.^23^^,^^24^

Using advanced natural language processing and machine learning on EHR data from a large multi-hospital health care system, we sought to develop a screening tool to aid timely identification of patients in a real-world HF population for whom ATTR-CM should be suspected as an underlying cause. The long-term goal is that such identification could serve as a flag to prompt appropriate clinical assessment and diagnostic testing.

## Methods

### Study population

Baylor Scott & White is a large integrated health care system in Texas, USA, with a diverse patient population and clinical setting, encompassing primary care community clinics, specialty cardiology practices, community hospitals, academic medical centers, and specialty cardiovascular hospitals. The study sample comprised patients aged ≥18 years with at least 1 Baylor Scott & White encounter in the EHR between April 1, 2020, and March 31, 2023, and an echocardiogram within 1 year before or after the index date of a qualifying diagnosis. Qualifying diagnoses, based on International Statistical Classification of Diseases–10th Revision–Clinical Modification (ICD-10-CM) codes, included HF (I50) and ATTR amyloidosis (E85) (Central Illustration, Supplemental Figure 1); light chain amyloidosis (E85.81) was excluded. Patients were classified as having raised clinical suspicion of ATTR-CM (E85 ICD-10-CM present) vs not (no E85 ICD-10-CM code). This case definition captured all patients for whom a clinical suspicion of ATTR-CM had been raised, rather than only patients with confirmed ATTR-CM diagnoses. The purpose was to cast a deliberately broad net for screening—given the issues with delayed and missed diagnoses in ATTR-CM—through the development of a model to identify patients for whom a similar level of suspicion should apply.

**Central Illustration:**
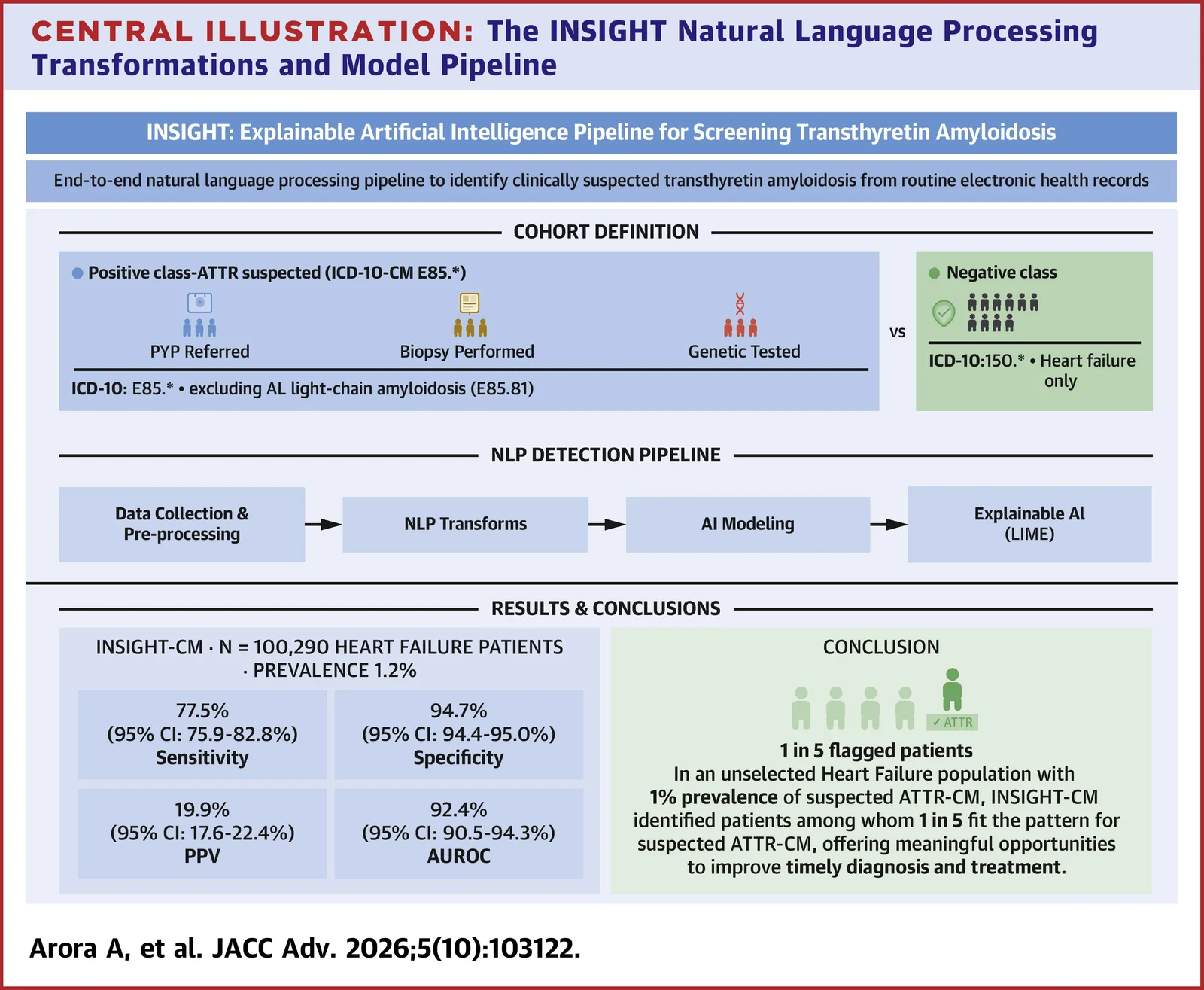
The INSIGHT Natural Language Processing Transformations and Model Pipeline AI = artificial intelligence; AL = amyloid light; ATTR = transthyretin amyloidosis; ICD-10-CM = International Statistical Classification of Diseases–10th Revision–Clinical Modification; LIME = local interpretable model-agnostic explanations; NLP = natural language processing; PYP = 99m-technetium-pyrophosphate imaging.

The Baylor Scott & White Research Institute Institutional Review Board approved the study.

### Data source

The primary data source was Baylor Scott & White’s Epic EHR. Key structured and unstructured data from 2020 to 2023 were extracted. Structured data included demographics, diagnosis, procedure codes, and health care encounters. The unstructured data included clinical notes such as chart notes, progress notes, and procedure notes. The procedure notes included observations from electrocardiogram or 2-dimensional transthoracic echocardiography reports. The data set also included laboratory result notes. The process of data collection and storage is summarized in the Central Illustration.

### Study outcomes

The main outcomes of interest were model performance metrics including sensitivity, specificity, PPV, negative predictive value (NPV), and F1 score (harmonic mean of PPV and sensitivity). Model interpretability was evaluated by identifying the key predictors driving classification.

### Text annotation

We developed a custom natural language processing pipeline to translate unstructured clinical documentation within the EHR into structured features for model development. Clinician annotators labeled clinical notes, echocardiograms, and electrocardiograms from a stratified sample of 500 patient records, enriched to ensure adequate representation of ATTR-CM cases alongside HF controls (250 ATTR-CM cases, 250 HF controls). Expert annotators collaboratively established labeling criteria for clinically relevant entities, including disease mentions, conditions, symptoms, echocardiographic findings, electrocardiographic findings, and their negations. This annotation was performed at the entity level, labeling clinical concepts such as diseases, conditions, symptoms, and their negations within each sentence and not at the case level. No note or patient was labeled as ATTR-CM or HF during this process, and the case status was defined solely by the presence of an ATTR amyloidosis ICD-10-CM code (E85) in the EHR. In this pipeline, manually annotated records were used to fine-tune a Clinical ModernBERT-based named-entity recognition model, which then auto-annotated the remaining clinical documentation. Expert annotators reviewed the auto-annotated output, corrections were applied, and the refined annotations were used to retrain the model. Duplicate entries were removed.

### Named entity recognition model fine-tuning

Building on this annotated data set, we fine-tuned a pretrained Clinical ModernBERT model to perform named entity recognition and automatically extract clinically relevant concepts from free-text notes. The model identified key entity categories, including disease mentions (eg, spinal stenosis), clinical conditions (eg, carpal tunnel), medications, procedures, transthoracic echocardiography findings (eg, apical sparing), and negations (eg, “no evidence of pericardial effusion”). This approach enabled systematic identification of both positive and negative clinical signals, preserving important contextual information. The fine-tuned named entity recognition model was subsequently applied to the full study population to generate structured features for downstream prediction. The model demonstrated high performance, with an overall accuracy of approximately 94% and consistently strong precision, recall, and F1-scores across clinically relevant entity categories (Supplemental Table 2). This approach enabled the scalable application of expert-derived annotations across a large real-world data set while preserving clinically meaningful representation of patient information.

Details of data preprocessing, deduplication, and the annotation process are provided in Supplemental Methods,^2^, ^3^, ^4^, ^5^, ^6^, ^7^, ^8^ Supplemental Tables 1, 3, Supplemental Figures 2, 3, and 5.

### Model development

The EHR data set was randomly divided into training (70%), validation (10%), and testing (20%) subsets, with preservation of the proportion of the minority class (patients with ATTR amyloidosis diagnosis codes) and majority class (patients with HF without ATTR amyloidosis diagnosis codes). This stratified split supported model development, validation, and unbiased performance assessment. Given the low prevalence of ATTR amyloidosis in the study population, class imbalance was addressed by incorporating class weights during training.

We used a transformer-based natural language processing approach centered on Clinical ModernBERT^25^ to represent unstructured clinical notes. Notes were first processed through a named entity recognition pipeline to extract recognized clinical entities such as symptoms, conditions, diseases, and their negations, producing a structured entity summary for each note; all other free-form text was discarded. Additionally, all the entities or terms that could directly reveal case status, such as including mentions of pyrophosphate scan, amyloid, amyloidosis, infiltrative cardiomyopathy, and ATTR, were excluded from the input feature set before model training to prevent label leakage.

These entity summaries were tokenized with the pretrained Clinical ModernBERT tokenizer and processed through the transformer encoder to obtain token-level hidden representations. Note-level embeddings were aggregated to the encounter level, and each patient was represented by all encounters preceding the ATTR amyloidosis diagnosis date. These patient-level representations served as input to a multilayer perceptron classifier consisting of fully connected layers followed by a sigmoid output for binary classification. The model was trained using the Adam optimizer and binary cross-entropy loss, and performance was evaluated on a held-out test set using area under the receiver-operating characteristic curve (AUROC), precision, recall, and F1-score. Structured data (ie, demographics, ICD-10-CM diagnosis codes, and procedure codes) were not used as model features; they were used only to construct the cohort and to report subgroup-stratified performance. Additional details are provided in the Supplemental Methods,^11^, ^12^, ^13^ Supplemental Table 2, and Supplemental Figure 2.

Using this unified natural language processing framework, the INSIGHT-CM model identified patients with ATTR amyloidosis diagnosis codes among patients with HF by transforming unstructured EHR text into structured entity representations, encoding them as contextual embeddings, and applying a downstream classifier for prediction (Supplemental Methods,^4^, ^5^, ^6^, ^7^ Supplemental Figures 2 and 3).

### Interpretability analysis

We conducted an interpretability analysis using local interpretable model-agnostic explanations (LIME), where predictions from both machine learning and deep learning models were explained by creating locally linear approximations. This approach highlighted the features that influenced individual outcomes, providing insight into the model’s decision-making process.

### Statistical analysis

For all descriptive analyses, categorical variables were reported as frequency and percentage, while continuous variables were summarized as mean and SD. Study metrics included sensitivity, specificity, PPV, NPV, and F1-score (the combination of PPV and sensitivity). Our models yielded continuous probabilities, which we dichotomized for the presence or absence of the conditions by thresholds that maximized the Youden index (defined as *J = Sensitivity + Specificity−1*, which identifies the optimal trade-off between sensitivity and specificity). We evaluated the model's performance by generating AUROC curves to analyze the trade-off between sensitivity and specificity and precision-recall curves to assess the balance between precision and recall, particularly for imbalanced datasets. Threshold adjustments were systematically applied to identify optimal cutoff points that maximize relevant performance metrics such as the F1 score. For each of these metrics, 95% CIs were obtained from bootstrap resampling with 1,000 iterations. Subgroup analyses were also conducted to assess the performance of the models across various groups based on race and ethnicity, sex, and ejection fraction. Analyses were conducted using Python with details in Supplemental Methods.^1^^,^^9^, ^10^, ^11^, ^12^, ^13^, ^14^, ^15^

### Model to identify patients with suspected ATTR amyloidosis with mixed phenotype

The same methods were applied to develop and evaluate a model to identify patients in whom ATTR-MP (ie, both cardiomyopathy and polyneuropathy) should be suspected and investigated (see ATTR-Mixed Phenotype Supplement section).

## Results

### Study cohort

A total of 100,290 patients with qualifying diagnosis codes and ≥1 encounters were identified from the Baylor Scott & White EHR between April 1, 2020, and March 31, 2023. Demographic and clinical characteristics are shown in Table 1. There were 1,108 patients with ATTR-CM diagnosis codes among 100,290 patients with HF diagnosis codes (mean age, 71.6 ± 14.3; female, 47.3%).

**Table 1:** Baseline Characteristics of Included Patients (N = 100,290)

Age, y	71.6 ± 14.3
Body mass index	30.5 ± 6.9
Sex	
Male	52,765 (52.6)
Female	47,516 (47.3)
Unknown	9 (0.1)
Race	
American Indian or Alaska Native	453 (0.5)
Asian	1,886 (1.8)
Black	18,208 (18.2)
Native Hawaiian or other Pacific Islander	230 (0.2)
White	76,417 (76.2)
Unknown	3,096 (3.08)
Ethnicity	
Hispanic	9,558 (8.7)
Non-Hispanic	89,960 (89.7)
Unknown	772 (0.7)
Marital status	
Married	47,730 (47.6)
Single	19,144 (19.1)
Smoking status	
Current	11,151 (11.1)
Former	37,726 (37.6)
Deceased status	
Alive	79,745 (79.5)
Deceased	20,545 (20.5)
Prior comorbidities	
Hypertension	82,795 (82.6)
Chronic kidney disease	44,929 (44.8)
Diabetes mellitus	46,333 (46.2)
Atherosclerotic cardiovascular disease	51,224 (51.1)
Atrial fibrillation	43,024 (42.9)
Myocardial infarction	26,918 (26.84)
Cardiomyopathy	30,278 (30.2)
Heart failure by LVEF	
HFpEF (LVEF ≥50%)	41,878 (41.7)
Unknown	34,945 (34.8)
HFrEF (LVEF ≤40%)	14,162 (14.2)
HFimpEF (previous LVEF ≤40%; follow-up LVEF >40%)	4,893 (4.87)
HFmrEF (LVEF 41%–49%)	4,413 (4.4)
Amyloidosis diagnosis	1,108 (1)

Values are mean ± SD or n (%).

HFimpEF = heart failure with improved ejection fraction; HFmrEF = heart failure with mildly reduced ejection fraction; HFpEF= heart failure with preserved ejection fraction; HFrEF = heart failure with reduced ejection fraction; LVEF = left ventricular ejection fraction.

### Model evaluation

In predicting suspected ATTR-CM (n = 1,108) among patients with HF (n = 100,290), INSIGHT-CM achieved a sensitivity of 77.5% (95% CI: 75.9-82.8), a specificity of 94.7% (95% CI: 94.4-95.0), a PPV of 19.9% (95% CI: 17.6-22.3), an NPV of 99.6% (95% CI: 99.5-99.7), and an F1 score of 31.7% (95% CI: 28.5-34.8) (Table 2). Figure 1 provides a summary of the performance of the best model, Clinical ModernBERT, and additional machine learning models. Key evaluation metrics, including accuracy, precision, recall, F1-score, NPV, specificity, and AUROC, are detailed in Table 2. Supplemental Table 2 highlights the metrics stratified by demographic characteristics.

**Table 2:** Performance Metrics for the Models Tested for INSIGHT-CM Based on the Strongest Threshold for Each

	PPV (%) (95% CI)	NPV (%) (95% CI)	Sensitivity (%) (95% CI)	Specificity (%) (95% CI)	F1 (%) (95% CI)	AUROC^a^ (%) (95% CI)	Screened Positive (%) (95% CI)
Clinical ModernBERT	19.9 (17.6-22.36)	99.6 (99.5-99.7)	77.5 (75.9-82.8)	94.745 (94.4-95.0)	31.71 (28.5-34.8)	92.4 (90.5-94.3)	6.5 (6.1-7.1)
BRNN	19.5 (17.4-21.2)	99.6 (98-100)	77.2 (75.5-81.5)	94.4 (90-97.5)	31.69 (30.5-33.5)	92.5 (90.5-94.1)	6.6 (6.4-7.2)
LGBM	17.1 (14.3-18.5)	99.8 (99.8-99.9)	91.9 (90.6-93.6)	92.4 (88.9-93.0)	28.8 (27.0-30.0)	97.2 (95.4-98.6)	9.1 (8.6-9.4)
HGBC	17.0 (15.4-18.4)	99.8 (99.7-99.9)	90.7 (89.7-92.8)	92.5 (91.3-94.4)	28.6 (26.5-29.6)	97.2 (94.5-97.7)	8.9 (8.5-9.3)
RFC	15.9 (9.2-17.2)	99.6 (99.5-99.7)	79.8 (78.2-81.2)	89.6 (88.9-92.0)	26.2 (22.7-28.7)	92.4 (91.2-94.3)	11.6 (11.1-12.0)
BBC	14.1 (12.6-15.7)	99.6 (99.6-99.7)	69.9 (67.8-70.9)	88.2 (85.7-88.7)	23.5 (16.7-24.8)	91.1 (90.6-93.7)	12.8 (12.3-13.2)
XGB	13.1 (9.0-15.1)	99.8 (99.8-100.0)	90.7 (90.4-93.5)	89.8 (84.4-90.4)	22.9 (15.9-23.4)	96.3 (94.2-97.5)	11.5 (11.1-12.0)

AUROC = area under the receiver-operating characteristic curve; BBC = Balanced Bagging Classifier; BRNN = Bidirectional Recurrent Neural Network; HGBC = Hist Gradient Boosting Classifier; LGBM = Light Gradient Boosting Classifier; NPV = negative predictive value; PPV = positive predictive value; RFC = Random Forest Classifier; XGB = Extreme Gradient Boosting Classifier.

a Based on continuous model.

**Figure 1:**
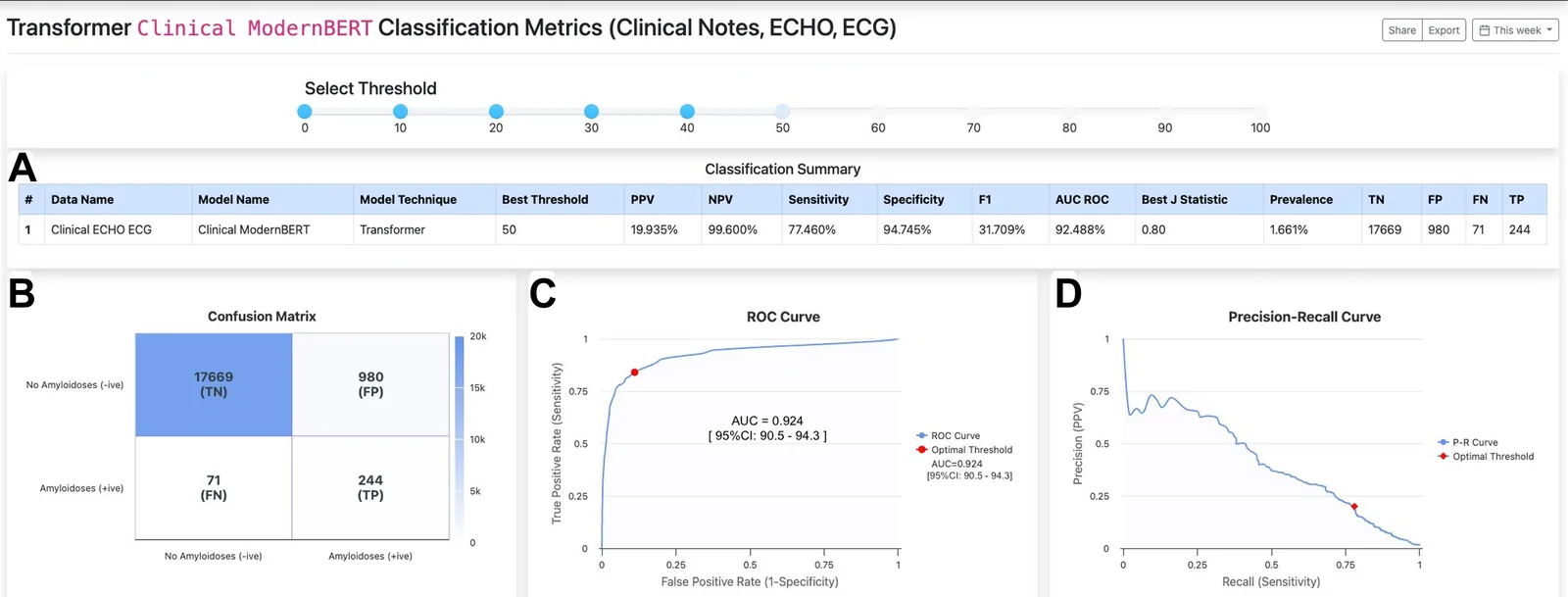
Performance Metrics of Machine Learning Models for ATTR-CM (A) Classification summary ATTR-CM. (B) Confusion matrix ATTR-CM. (C) AUROC curve ATTR-CM. (D) Precision–recall curve ATTR-CM. ATTR-CM = transthyretin amyloidosis cardiomyopathy; AUROC = area under the receiver-operating characteristic curve; ECG = electrocardiogram; ECHO = echocardiogram; FN = false negative; FP = false positive; NPV = negative predictive value; PPV = positive predictive value; TN = true negative; TP = true positive.

### Model interpretability

LIME-based interpretability analysis revealed key features that machine learning models leveraged for identification of suspected ATTR-CM. The top predictors were carpal tunnel, reduced longitudinal strain, fatigue, mass, cardiomyopathy, orthopnea, arm swelling, paroxysmal dyspnea, weight loss, and chronic kidney disease (Figure 2).

**Figure 2:**
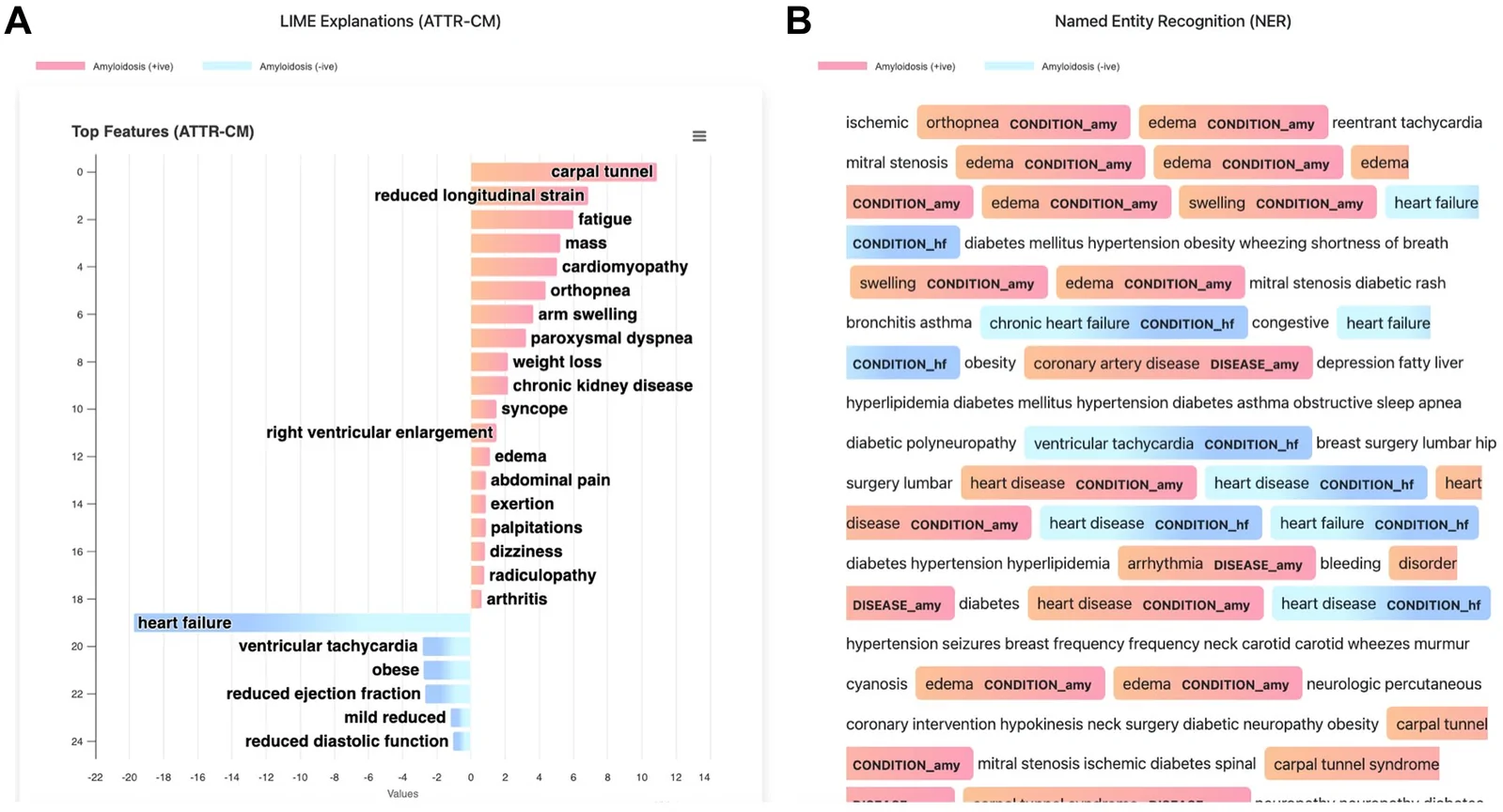
Top Predictors by LIME for ATTR-CM (A) LIME model explanations ATTR-CM. (B) Named entity recognition ATTR-CM. LIME = local interpretable model-agnostic explanations; other abbreviation as in Figure 1.

## Discussion

We developed a novel deep learning approach to identify patients with suspected ATTR-CM from unstructured notes, electrocardiogram, and echocardiographic data. INSIGHT-CM demonstrated high sensitivity and specificity. PPV was 19.9% in the context of ∼1% suspected ATTR-CM prevalence in the general HF population considered. This is substantially higher than the <1% PPV previously achieved in this Baylor Scott & White population applying the Mayo Clinic Simple Score,^12^ as well as the 3.4% PPV reported for the ALARM natural language processing-based model in a similar context.^23^ Additionally, the incorporation of LIME in INSIGHT identifies the key text-derived predictors underlying model decisions; interpretability could help clinicians validate the rationale behind each decision and further support clinical decision-making. Finally, INSIGHT-CM was developed to be adaptable across health care system EHRs.

Once ATTR-CM is diagnosed, available treatments have shown meaningful reductions in morbidity and mortality and/or preservation of functionality.^26^, ^27^, ^28^, ^29^, ^30^ Diagnosis, however, remains a substantial challenge due to diverse clinical manifestations, with many patients seeing multiple cardiologists and being treated for misdiagnoses before receiving an accurate diagnosis.^31^ Since patients are likely to present to nonspecialists with their initial symptoms, and since treatments offer the greatest benefits when initiated early in the disease course,^32^^,^^33^ an operational challenge is to develop systems that facilitate early detection in nonspecialist settings.^34^ Machine learning can help identify complex patterns in data, turning seemingly insignificant individual features into predictive indicators when combined.^35^ Moreover, integrating natural language processing with machine learning enables unstructured EHR data to be processed with the precision of manual curation but more efficiently and cost-effectively.

Previous studies similarly developing models to predict ATTR-CM have used E85 ICD-10 codes to define the cases vs controls.^18^^,^^24^ Although they considered the ICD-10 codes as proxies for a diagnosis (acknowledging the misclassifications that are inevitable among the study limitations^18^^,^^24^), rather than indicating a suspected diagnosis as we did, the use of the same overall approach to identifying cases and controls enables comparison. The ALARM study^23^^,^^24^ was most similar to INSIGHT, developing a machine learning model for predicting amyloidosis from unstructured EHR data. It achieved an AUROC of 0.88, sensitivity of 74%, specificity of 87%, PPV of 3.4%, and NPV of 99%, with an amyloidosis prevalence of 1%. INSIGHT, trained on a cohort with similar prevalence, achieved a sensitivity of 77.5%, specificity of 94.7%, PPV of 19.9%, and NPV of 99.6% for predicting ATTR-CM. A second study used a random forest model trained on medical claims data from 2,142 patients with HF and ATTR amyloidosis that demonstrated accuracy, sensitivity, and specificity of 87%, PPV of 88%, and NPV of 86%, but notably in a data set in which ATTR amyloidosis cases and nonamyloid HF controls were matched one-to-one (ie, 50% prevalence).^18^ How models developed in study cohorts with such artificially high ATTR amyloidosis prevalence perform in real-world populations is unknown.

PPV is dependent on disease prevalence; at higher prevalence, true positives make up a larger proportion of predicted cases, whereas at lower prevalence, more false positives are introduced. When considering what constitutes an acceptable PPV, context is critical, with relevant factors including patients’ health status, treatability of the condition, immediate and long-term burdens on the health care system, the psychological effect on patients, over- vs under-application of diagnostic procedures, and the risks of premature vs inappropriately delayed diagnosis.^36^ In the case of ATTR amyloidosis, a condition with frequently missed^37^ and delayed diagnosis,^5^^,^^31^ with a high mortality risk^1^^,^^2^ but amenability to life-extending treatments,^26^, ^27^, ^28^, ^29^, ^30^ and for which noninvasive diagnostic testing is available,^38^ screening tools that can be applied to the general HF population and identify patients among whom 1 in 5 fit patterns of suspected ATTR-CM offer meaningful clinical utility. Both external validation and prospective testing of INSIGHT are needed to determine whether it delivers on this potential. In context, the PPV of 19.9% observed here should not be considered a barrier to INSIGHT progressing to those steps.

Looking at performance measures that are less dependent on prevalence, previously published models for predicting ATTR-CM have reported sensitivity and specificity ranging from 18% to 95% and 75% to 99% in machine learning models that defined ATTR-CM cases based on ICD-9/10 codes,^14^^,^^15^^,^^18^^,^^19^ and 75 to 93% and 66% to 85% in deep learning models that defined ATTR-CM cases through diagnostic tests.^17^^,^^39^ Our model exhibited sensitivities and specificities at the high end of these ranges. Our model leverages annotated unstructured clinical, echocardiogram, and electrocardiogram notes to raise clinical suspicion. The data set includes clinical observations, patient histories, and diagnostic details. Trained on a large HF cohort (n = 100,290), our model demonstrates broad applicability. Additionally, negation handling improves accuracy by distinguishing affirmed from negated conditions, enhancing interpretability.

Delays in diagnosing ATTR-CM often result from patients’ nonspecific symptoms.^5^^,^^22^^,^^40^ Carpal tunnel syndrome is a common early sign, potentially appearing 5 to 10 years before an ATTR amyloidosis diagnosis.^41^^,^^42^ Data from a previous natural language processing-based machine learning model predicting ATTR-CM in patients with HF shows that atrial fibrillation, hypertension, paraproteinemia, chronic kidney disease, joint disorders, soft tissue disorders, and diastolic HF are other clinical characteristics that can occur with ATTR-CM.^22^ These characteristics have also been identified in the literature.^43^, ^44^, ^45^, ^46^ Our LIME results yielded similar characteristics with the addition of fatigue, orthopnea, arm swelling, paroxysmal dyspnea, and weight loss. These additional symptoms enrich the diagnostic profile and improve precision in distinguishing patients in whom ATTR amyloidosis should be suspected. Hence, collectively integrating INSIGHT-CM into routine clinical workflows may improve early detection of ATTR-CM.

### Study limitations

First, the data were derived from a single health care system, which may limit generalizability. Second, these are retrospective observational data; hence, there may be residual and unmeasured biases or confounding factors influencing our results. For example, recent studies suggest women are underdiagnosed for ATTR-CM, due to factors such as testing inertia, and failure to account for sex differences in body size when applying criteria for left ventricular hypertrophy.^47^ Third, some misclassification between the suspected ATTR amyloidosis and HF groups will have occurred. While we cast a deliberately wide net for suspected ATTR amyloidosis cases using ICD-10-CM codes, this can introduce inaccuracies;^48^^,^^49^ additionally, any undiagnosed ATTR amyloidosis cases that failed to raise clinical suspicion that would have resulted in an E85 ICD-10-CM code being assigned will have been misclassified. Fourth, the conversion of unstructured data into a structured format via natural language processing may result in the incomplete detection of certain variables, which could impact the accuracy and completeness of the data.^50^ Lastly, the proportion of Asians and Hispanics was relatively small, and subgroup analysis could not be performed.

## Conclusions

We developed a machine learning model INSIGHT-CM to identify patients for whom further evaluation for ATTR-CM should be considered. INSIGHT-CM demonstrated high predictive performance to identify suspected ATTR-CM among patients with HF. Incorporating INSIGHT-CM into EHR systems could flag patients who may benefit from further evaluation for ATTR amyloidosis, and the inclusion of patient-level LIME results enables clinicians to validate the rationale behind each decision. Future research should include prospective studies examining whether such use of INSIGHT-CM enables more timely diagnosis and intervention for ATTR-CM, improving optimization of care and patient outcomes.

Perspectives **COMPETENCY IN MEDICAL KNOWLEDGE:** From an unselected HF cohort (1% suspected ATTR-CM prevalence) in a large integrated health care delivery system, we developed an explainable artificial intelligence INSIGHT model to identify patients who should be suspected of having ATTR-CM. Using routinely collected data from the EHR, INSIGHT identified patients among whom 1 in 5 fit the pattern for suspected ATTR-CM and stand to benefit from being evaluated in that light. As such, INSIGHT offers the opportunity to screen full populations of HF patients using existing EHR data to identify actionable groups who should be considered for diagnostic testing. This may enable earlier diagnosis and treatment.

**TRANSLATIONAL OUTLOOK:** Further work is needed to develop strategies to make INSIGHT easily adaptable for use with other health care systems’ data, both for external validation and ultimately to facilitate adoption. Additionally, prospective studies are needed to determine how the model functions in real time, and how its use can be most effectively integrated into clinical workflows.

### Data and Code Availability

Data available on reasonable request to the authors. INSIGHT code available at https://github.com/bswh-research/INSIGHT.

## Funding support and author disclosures

This work was funded by AstraZeneca and generously supported by a philanthropic gift from Ashley and Greg Arnold. Dr Khan has received fees from Bayer and Novartis. Dr Gottlieb is a consultant/advisor, and/or has received grants or speaking/lecture fees from Alnylam Pharmaceuticals Inc, AstraZeneca, BridgeBio, Eli Lilly and Company, Pfizer, and Johnson & Johnson. Dr Masri has received research grants from Pfizer, Ionis, Attralus, Cytokinetics, and Janssen; and consulting fees from BridgeBio, Pfizer, Ionis, Lexicon, Attralus, Cytokinetics, BMS, Alnylam, Haya, Edgewise, Alexion, Akros, Prothena, BioMarin, Lexeo, AstraZeneca, Rocket, and Tenaya. Dr Priest has received grants from AstraZeneca, Boehringer Ingelheim, CSL Vifor, Eli Lilly, Owkin, and Recordati. Drs Papas and Venditto are employed by AstraZeneca with ownership in AZ stock. Dr Butler is a consultant to Abbott, American Regent, Amgen, Applied Therapeutic, AskBio, Astellas, AstraZeneca, Bayer, Boehringer Ingelheim, Boston Scientific, Bristol Myers Squibb, Cardiac Dimension, Cardior, CSL Behring, CVRx, Cytokinetics, Edwards, Element Science, Faraday, Foundry, Innolife, Impulse Dynamics, Imbria, Inventiva, Ionis, Lexicon, Lilly, LivaNova, Janssen, Medtronics, Merck, Occlutech, Owkin, Novartis, Novo Nordisk, Pfizer, Pharmacosmos, Pharmain, Prolaio, Regeneron, Renibus, Roche, Salamandra, Sanofi, SC Pharma, Secretome, Sequana, SQ Innovation, Tenex, Tricog, Ultromics, Vifor, and Zoll. All other authors have reported that they have no relationships relevant to the contents of this paper to disclose.
