# Supplementary material for: INSIGHT Model: Explainable Artificial Intelligence for Real-World EHR-Based Screening of Transthyretin Amyloidosis Cardiomyopathy
Source: JACC Adv. 2026 Aug 26;5(10):103122. doi: 10.1016/j.jacadv.2026.103122 (PMC13699708; doi:10.1016/j.jacadv.2026.103122)

Online Supplemental Content

eMethods

1. **Software and Libraries**
2. **Data Preprocessing**
3. **Data Deduplication**
4. **Text Annotation**
5. **Model Annotation**
6. **Negation Filtering**
7. **Text Summarization**
8. **Data Compression**
9. **Model Classification Metrics and Interpretability Dashboard**
10. **Advanced Computational Environment**
11. **Deep Learning Model Architecture and Workflow**
12. **Model Development**
13. **Comparator Models**
14. **Training and Hyperparameter Tuning**
15. **Natural Language Processing Transformations**
16. **Topic Modeling**

eReferences

eFigure 1. Summary of Study Design

eFigure 2. INSIGHT NLP Model Pipeline and Model Development

eFigure 3. NER Annotation Process

eFigure4. NER for Echocardiogram and ECG Data

eFigure5. Negation Filtering

eFigure6. Topic Modeling

eTable 1. Deep Learning Algorithm Summary

eTable 2. Demographic Characteristics with Corresponding Classification Metrics for Cohort (ATTR- CM)

eTable 3. Named Entity Recognition Model Performance Metrics

eTable4. Comparator Models Overview

eTable5. Hyperparameter Details

eTable6. Training and Evaluation Settings

ATTR-Mixed Phenotype Supplement

eMethods.

1. Software and Libraries

All analyses were conducted in a Python 3.12 environment. The following libraries were used:

- **Pandas (version 2.2.2)** for data manipulation
- **NumPy (version 1.26.4)** for numerical computations
- **SciPy (version 1.13.1)** for scientific computing
- **Scikit-learn (version 1.5.2)** for data splitting, evaluation metrics, and class weighting
- **PyTorch (version 2.2.2)** for transformer-based model inference
- **Transformers (version 4.44.2)** for implementation of the pre-trained Clinical ModernBERT model
- **TensorFlow (version 2.16.1)** and **Keras (version 3.3.3)** for development of the downstream multilayer perceptron classifier
- **PyArrow (version 17.0.0)** for efficient data storage and Parquet file handling
- **SQLAlchemy (version 2.0.36)** for database interactions
- **pyodbc (version 5.1.0)** for connectivity to SQL-based data sources
- **TopicWizard (version 1.1.2)** for visualization and exploratory analysis

These libraries were selected for their reliability, scalability, and widespread use in clinical natural language processing and machine learning workflows. Pandas and NumPy enabled efficient handling of large-scale EHR data, while PyTorch and the Transformers library supported extraction of contextual embeddings using the pre-trained Clinical ModernBERT model. TensorFlow and Keras were used to develop the downstream multilayer perceptron classifier for prediction. Scikit-learn facilitated model evaluation and handling of class imbalance

through class weighting. PyArrow enabled efficient data storage and retrieval, and database connectivity was managed through SQLAlchemy and pyodbc.

2. Data Preprocessing

The clinical notes dataset, already compressed in batches, was preprocessed in a batch-wise manner. As illustrated in **eFigure 2, 3 and 4**, the NLP preprocessing workflow involves decompressing each batch parallelly and applying the preprocessing methods described in the following sections. We conducted data filtering based on clinically significant events, focusing on a 12-month window preceding the initial transthyretin (ATTR) amyloidosis diagnosis as identified by relevant International Classification of Diseases, Tenth Revision, Clinical Modification (ICD-10-CM) codes. This temporal filtering isolated critical clinical information leading up to the diagnosis. The filtered dataset then went through an intensive text preprocessing phase for NLP. This involved tokenizing the text, removing stop words (is, are, of, on etc.) and punctuation to eliminate the noise and standardize the clinical text. Additionally, we expanded abbreviations to their full medical terms such as AFIB - Atrial Fibrillation, HLD - hyperlipidemia etc. to ensure the data accurately reflected clinical meanings. We used SpaCy (1) for the NLP transformations illustrated in **eFigure 2, 3 and 4** of the NLP Pipeline. Thus, this prepared the dataset for the deduplication phase.

The classifier did not operate on free-text notes. After named entity recognition (NER), all text other than recognized clinical entities (symptoms, conditions, diseases, and their negations) was discarded, and only this structured entity set was retained as a summary of each note. Narrative documentation of diagnostic suspicion, such as planned PYP imaging, monoclonal screening, or

phrasing such as “consider amyloid,” is therefore not represented in the model input. Note-level entity summaries were aggregated to the encounter level, and each patient was represented by the entities from all encounters preceding the ATTR amyloidosis diagnosis ICD-10-CM date. The only residual channel for the outcome would be an explicit amyloidosis disease entity, and LIME did not rank amyloidosis terms among the top predictors, indicating that prediction was driven by phenotypic signs rather than by the diagnostic label.

3. Data Deduplication

Data duplication often arises from multiple sources of clinical documentation, such as repeated entries in patient records, updates from different healthcare providers, or overlapping information between systems. The practice was prevalent across all levels of healthcare professionals regardless of their training or experience and was equally common among both those who had authored previous notes and those referencing earlier documentation. This redundancy can muddy the data, making it harder to extract accurate clinical insights. To ensure the dataset's quality and relevance, we implemented a robust text deduplication process that began with identifying duplicate entries using text similarity techniques. This step ensured the detection of identical and near-identical records. Redundant text was systematically eliminated, with a focus on consolidating records while preserving unique clinical insights. Finally, the deduplicated data was formatted for downstream NLP workflows, minimizing noise in the raw clinical text.

4. Text Annotation

We developed a custom named entity recognition (NER) pipeline to convert unstructured clinical text into a structured representation (eFigure 3, 4, and 5). Expert-annotated notes were used to fine-tune a transformer-based Clinical ModernBERT model, which then labeled each note with entity tags including DISEASE, CONDITION, NORMALCY, and NEGATION. Because these entities, rather than the free-form text, formed the input to the downstream model, NER served two purposes: it identified clinically relevant features, and it removed any text that was not a recognized entity. eFigure 3 illustrates the annotation process, showing how unstructured clinical text was labeled and transformed into a structured JSON format.

Each tag captures a distinct type of clinical information:

DISEASE refers to a named diagnosis or established disease, for example heart failure, hypertension, aortic stenosis, atrial fibrillation, cardiac amyloidosis, or carpal tunnel syndrome.

CONDITION refers to a sign, symptom, or clinical finding that is not itself a formal diagnosis, for example, dyspnea, edema, lightheadedness, spinal stenosis, paresthesia, or increased wall thickness, or pericardial effusion.

NORMALCY refers to an explicit statement that a structure or parameter is normal or within normal limits, for example, normal left ventricular systolic function, normal breathing sounds, normal sinus rhythm, etc.

NEGATION refers to an entity that is explicitly denied or absent, which allows the model to separate affirmed from negated findings, for example no chest pain, denies syncope, or no evidence of ischemia. The role of NEGATION is described further in the next section.

The contrast is the basis for discrimination. A control patient is characterized by heart failure entities in an ischemic or hypertensive context with the multisystem red flags explicitly negated,

whereas an ATTR-positive patient carries the same heart failure entities plus affirmed red-flag entities such as carpal tunnel syndrome, polyneuropathy, and disproportionate wall thickness, often alongside normalcy statements (for example, normal atrial size/pressure) that argue against a competing cause. Expert annotation was used to label these entities in a subset of notes in order to train and validate the NER model and to support feature extraction; it was not used to define case status. Case status was defined at the patient level by the presence of an ATTR amyloidosis ICD-10-CM code (E85; excluding light-chain amyloid E85.81), as described in the Methods in manuscript.

We extended the NER system to echocardiographic data to extract entities such as ANATOMY, ECHO_MEASURE, ECHO_FINDING, UNIT, and NORMALCY. For example, as shown in eFigure 5, the system captures features of the form ANATOMY plus ECHO_MEASURE plus ECHO_FINDING, producing phrases such as "Right Ventricle Moderately Enlarged." We similarly enhanced the system for ECG data to extract entities such as ECG_MEASURE, ECG_FINDING, and CARDINAL, capturing features of the form CARDINAL plus ECG_FINDING, producing phrases such as "10 Atrial Fibrillation Episodes." This yields a more detailed and structured representation of the clinical record.

Two small things to confirm: the examples are illustrative, so check that the specific NORMALCY phrasing for the ATTR column (non-obstructive coronaries, low or normal blood pressure off antihypertensives) matches how your annotators actually tagged those, since those are sometimes captured as NEGATION rather than NORMALCY depending on the source sentence.

To efficiently annotate the large dataset, we adopted a semi-automated approach. Initially, a subset of the clinical notes was manually annotated by domain experts, ensuring accurate

identification of critical entities. These annotations were then used to fine-tune the Clinical ModernBERT-based NER model, which subsequently auto-annotated the remaining notes. The NER transformation process is illustrated in eFigure 2 and 5. The auto-annotated data was iteratively refined through cycles of review and re-annotation, with the model improving its performance after each iteration. Finally, an expert reviewed the fully annotated dataset, conducting quality checks to ensure consistency and accuracy. The final model demonstrated an overall accuracy of approximately 94%, with strong precision, recall, and F1-score across entity categories (eTable 3).

This iterative annotation process was critical for building a robust NER model. It not only accelerated the annotation of a large dataset but also maintained high-quality entity recognition

Model Annotation

Each patient had multiple clinical encounters, and each encounter generated various note types including progress notes, provider notes, order narratives, echocardiogram impressions and findings, electrocardiogram narratives, discharge instructions, procedure notes, and cardiology and neurology consults. The total volume of documents processed therefore substantially exceeded the number of patients.

All annotation was performed at the entity level, where each sentence was tagged for clinically relevant concepts (diseases, conditions, symptoms, echocardiographic findings, electrocardiographic findings, and negations), and not at the case level. No document was classified as ATTR-CM or heart failure; these entity tags were subsequently aggregated to generate structured feature representations for each patient.

Of the 167,418 clinical notes processed (median 2.4 notes per patient), 9,305 (5.6%) contained at least one explicit mention of ATTR-CM and 3,425 (2.1%) contained at least one explicit mention of ATTR-MP. Of the 293,215 echocardiogram reports processed (median 3.2 per patient), 2,690 (0.91%) contained entity features associated with ATTR-CM and 892 (0.31%) with ATTR-MP. Of the 336,127 electrocardiogram narratives processed (median 4 per patient), 4,305 (1.3%) contained entity features associated with ATTR-CM and 1,596 (4.7%) with ATTR-MP.

6. Negation Filtering

We used specialized NLP techniques such as dependency parsing to detect negations in the clinical text, allowing the model to distinguish between affirmed and negated medical statements. For instance, phrases like ‘no evidence of shortness of breath’, ‘no chest pain’, ‘denies history of arrhythmia’ etc. were accurately flagged as negations. We applied context-aware pattern recognition that identifies negation cues and their scope within sentences. By implementing a systematic approach to negation filtering, we ensured that the extracted data accurately reflected the patient's clinical status, enhancing the reliability of downstream predictive modeling and analysis. **eFigure 4** illustrates the negation handling and filtering process.

7. Text Summarization

After the NER step, the entities extracted from each note were assembled into a concise, structured summary of the patient's clinical record. Only recognized entities such as DISEASE, CONDITION, ECHO_FINDING, ECG_FINDING, NORMALCY, and NEGATION were retained, and all other free-form text was discarded. Entities or terms that could directly reveal case status, such as including mentions of pyrophosphate (PYP) scan, amyloid, amyloidosis, infiltrative cardiomyopathy, and ATTR, were excluded from the input feature set before model training to prevent label leakage.

The result was a compact representation that preserved the clinically relevant diagnostic and contextual information while removing noise and redundant documentation.

These entity summaries were the final step of unstructured-data preprocessing and served as the input to feature extraction. Each summary was encoded with the pre-trained Clinical Modern BERT model, which produced contextual embeddings that capture semantic and contextual relationships among the retained entities. The contextual embeddings from Clinical Modern BERT were the only representation passed to the downstream classifier. Because these embeddings were computed on focused, noise-reduced summaries rather than raw notes, the resulting features were cleaner and more directly tied to interpretable clinical entities.

8. Data Compression

Given the dataset's extensive size covering 100,290 patients and nearly 1.5 million encounter records. We implemented a fast batch querying process to efficiently manage and retrieve the data. Each batch contained 1,000 encounter identifiers, resulting in approximately 1,500 batches being processed. During the querying process, the data was dynamically compressed on the fly

using Parquet, a columnar storage format that offers high compression rates. This compression reduced storage requirements while simultaneously improving query performance. The overall batch dataset volume after compression was around 8.5 GB. The compressed data was securely stored in Microsoft Azure Blob Storage, which seamlessly supports Parquet files. This setup allowed for efficient data retrieval and analysis while ensuring that the data's structure and granularity were preserved. By minimizing strain on database resources and optimizing overall performance, this approach provided a scalable and efficient solution for managing large-scale clinical data, making it ready for downstream machine learning applications.

9. Model Classification Metrics and Interpretability Dashboard

We used Highcharts, Node.js, Bootstrap, CSS, HTML, and JavaScript to build an interactive dashboard for studying machine learning metrics like confusion matrix, receiver operating characteristic (ROC) curves, PR curves, and LIME based top feature analysis and model interpretability dashboard.

- **Highcharts** helped create interactive charts and graphs to explore model performance.
- **Node.js** handled the server-side rendering data to the dashboard.
- **Bootstrap** made the dashboard responsive, ensuring it looks good on any device with ready-to-use components.
- **CSS** was used for styling the dashboard
- **HTML** structured the content and organized the layout for each metric.
- **JavaScript** added interactivity, allowing dynamic updates and user interactions with the dashboard.

These tools worked together to create an easy-to-use and functional platform for analyzing machine learning model results.

10. Advanced Computational Environment

For the deep learning phase, we utilized a high-performance Apple M3 Max Mac system equipped with a 40-core GPU, 128 GB RAM, and 16 CPU cores. This computational setup, combined with the TensorFlow Metal kernel with keras wrapper, provided the processing power needed for efficient training and experimentation.

11. Deep Learning Model Architecture and Workflow

We developed a transformer-based natural language processing (NLP) framework to model unstructured clinical text. This approach leverages a pre-trained Clinical ModernBERT architecture to generate contextualized representations of clinical narratives, enabling downstream supervised learning for ATTR amyloidosis prediction.

12. Model Development

Clinical text was encoded using contextual embeddings derived from a pre-trained Clinical ModernBERT model (eFigure 2). Input text was tokenized using the model-specific tokenizer and processed through the transformer encoder to obtain token-level hidden representations. To derive a fixed-length representation for each clinical note, masked mean pooling was applied

over the sequence dimension, producing a 768-dimensional embedding vector while excluding padded tokens via the attention mask.

These embeddings served as input to a downstream multilayer perceptron (MLP) classifier. The classifier architecture consisted of fully connected layers (256 and 768 units) with ReLU activation and dropout regularization, followed by a sigmoid output layer for binary classification. The model was trained using the Adam optimizer with binary cross-entropy loss.

Unit of analysis and model inputs. The unit of prediction is the patient. The classifier did not use free-text notes; after named entity recognition, only recognized clinical entities (symptoms, conditions, diseases, and their negations) were retained as a structured summary of each note. Each note-level entity summary was encoded with Clinical ModernBERT using masked mean pooling to produce a 768-dimensional vector. Note-level summaries were aggregated to the encounter level, and each patient was represented by all encounters preceding the ATTR amyloidosis diagnosis ICD-10-CM date. Structured data (demographics, ICD-10-CM diagnosis codes, and procedure codes) were not used as model features; they were used only to construct the cohort and to report subgroup-stratified performance (eTable 2). The predicted outcome was the presence of an ATTR amyloidosis diagnosis (ICD-10-CM E85) at the patient level.

Given the low prevalence of ATTR amyloidosis in the study population, class imbalance was addressed using inverse-frequency class weighting during training. Model performance was evaluated on a held-out test set using area under the receiver-operating characteristic curve (AUROC), precision, recall, and F1-score.

13. Comparator Models

In addition to the primary model, we evaluated logistic regression (LOG), a balanced random forest classifier (BRFC), a balanced bagging classifier (BBC), a random under-sampling boosting classifier (RUS), and extreme gradient boosting (XGB). All comparators used the same patient-level feature set (Clinical ModernBERT embeddings) and the same outcome (presence of an ATTR ICD-10-CM code at the patient level). Class imbalance was handled by inverse-frequency class weights for LOG and XGB, and internal balancing for BRFC, BBC, and RUS. Hyperparameters were tuned by grid search with k-fold cross-validation (k=4) on the training set. The operating threshold for each model was selected by the maximum Youden J statistic, and all metrics in Table 2-MP are reported at that threshold. Confidence intervals were obtained by bootstrap with 1000 resamples.

14. Training and Hyperparameter Tuning

The downstream multilayer perceptron (MLP) classifier was trained using the Adam optimizer with an initial learning rate of 1×10^{-3}. No explicit learning rate decay schedule was applied; instead, model convergence was monitored through validation loss.

A batch size of 64 was used, and the model was trained for up to 75 epochs. Early stopping was implemented based on validation loss, with training terminated if no improvement was observed for five consecutive epochs, thereby preventing overfitting.

Binary cross-entropy was used as the loss function for optimization. Model performance was evaluated using area under the receiver-operating characteristic curve (AUROC), precision (positive predictive value), recall (sensitivity), and F1-score. The F1-score was included to provide a balanced assessment of performance in the presence of class imbalance.

To further address class imbalance, inverse-frequency class weights were applied during training, increasing the contribution of minority class samples to the loss function.

Training progress was monitored using TensorBoard, and model checkpoints were used to retain the best-performing model based on validation loss.

15. Natural Language Processing Transformations

i. Transformer-Based Text Representation

We employed a transformer-based embedding approach to convert unstructured clinical text into dense vector representations. Clinical ModernBERT generates contextual embeddings by encoding bidirectional dependencies within the input sequence, allowing each token representation to incorporate global context.

To accommodate long-form clinical notes, sequences were truncated to a maximum length of 2,048 tokens. Token-level embeddings from the final hidden layer were aggregated using masked mean pooling, resulting in a fixed-length vector representation that preserves semantic and contextual information.

ii. Downstream Feature Learning

The resulting embeddings provided high-dimensional, context-aware representations of clinical notes and were used as input features for the downstream MLP classifier.

16. Topic Modeling

Instead of traditional topic modeling approaches, we leveraged transformer-based representation learning to capture latent structure within clinical text. Clinical ModernBERT generates contextualized embeddings that encode semantic and syntactic relationships across the entire input sequence, allowing the model to capture complex clinical patterns without explicit topic modeling.

The contextual embeddings produced by Clinical ModernBERT inherently represent latent themes and relationships within the data, effectively replacing classical techniques such as Latent Semantic Indexing (LSI) and Non-Negative Matrix Factorization (NMF). These embeddings were subsequently used as input features for downstream classification, enabling the model to learn clinically relevant patterns directly from the data.

This approach allows for richer representation of clinical language, as embeddings incorporate long-range dependencies and contextual meaning that cannot be captured by frequency-based methods. The overall transformation pipeline is illustrated in eFigure 6.

eReferences:

1. Clinical ModernBERT: An efficient and long context encoder for biomedical text

arXiv:2504.03964

eFigure 1: Summary of study design.

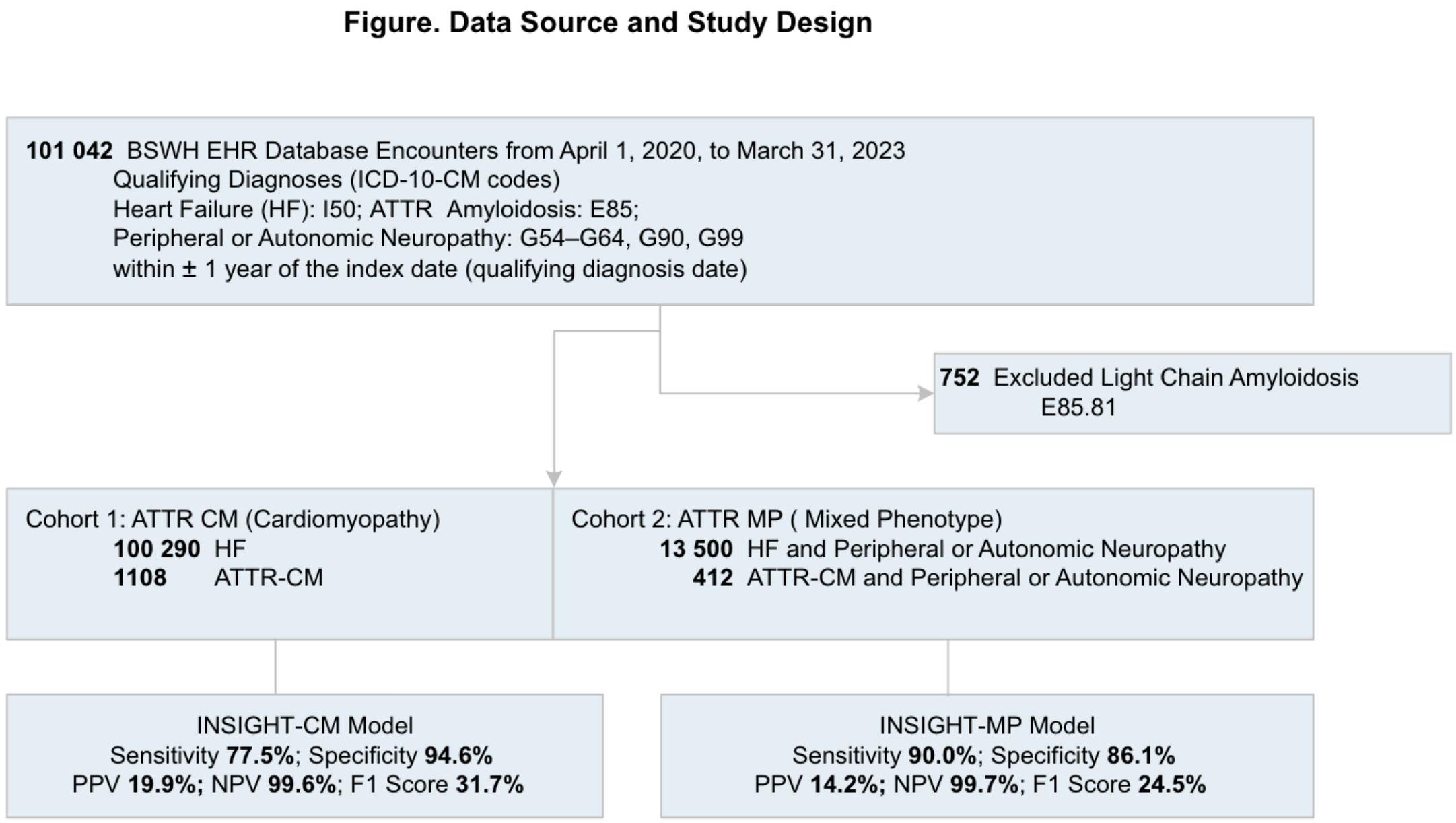

eFigure 2: INSIGHT NLP Model Pipeline and Model Development

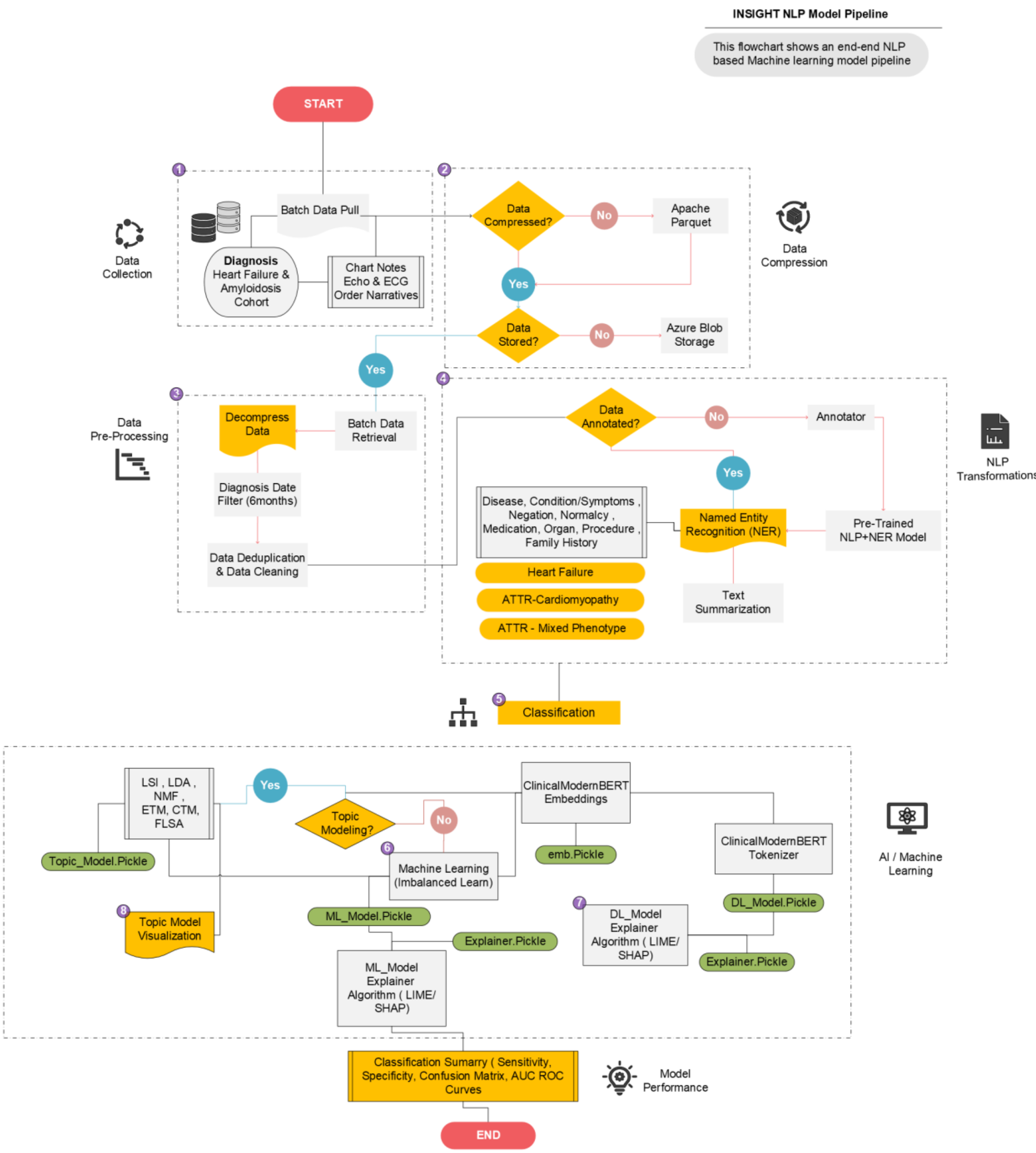

eFigure 3: NER Annotation process

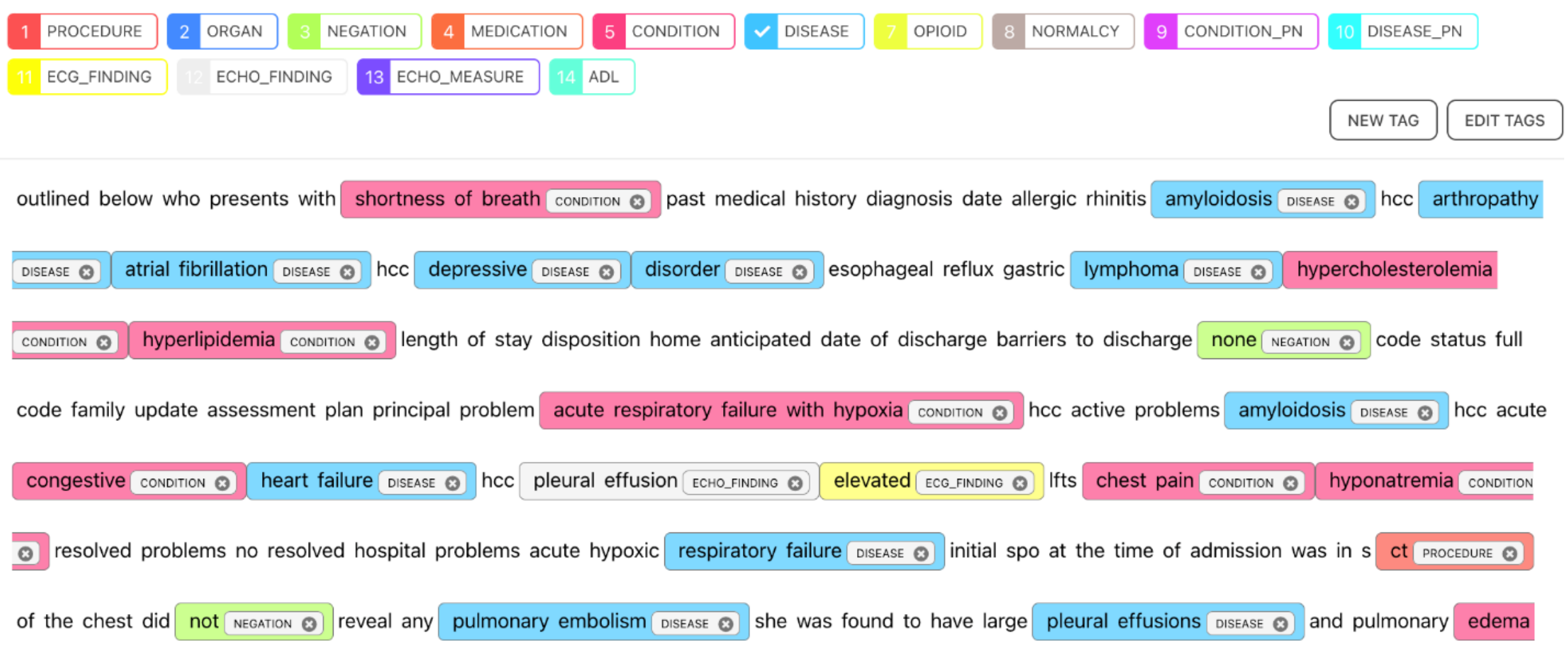

eFigure 4: Negation Filtering

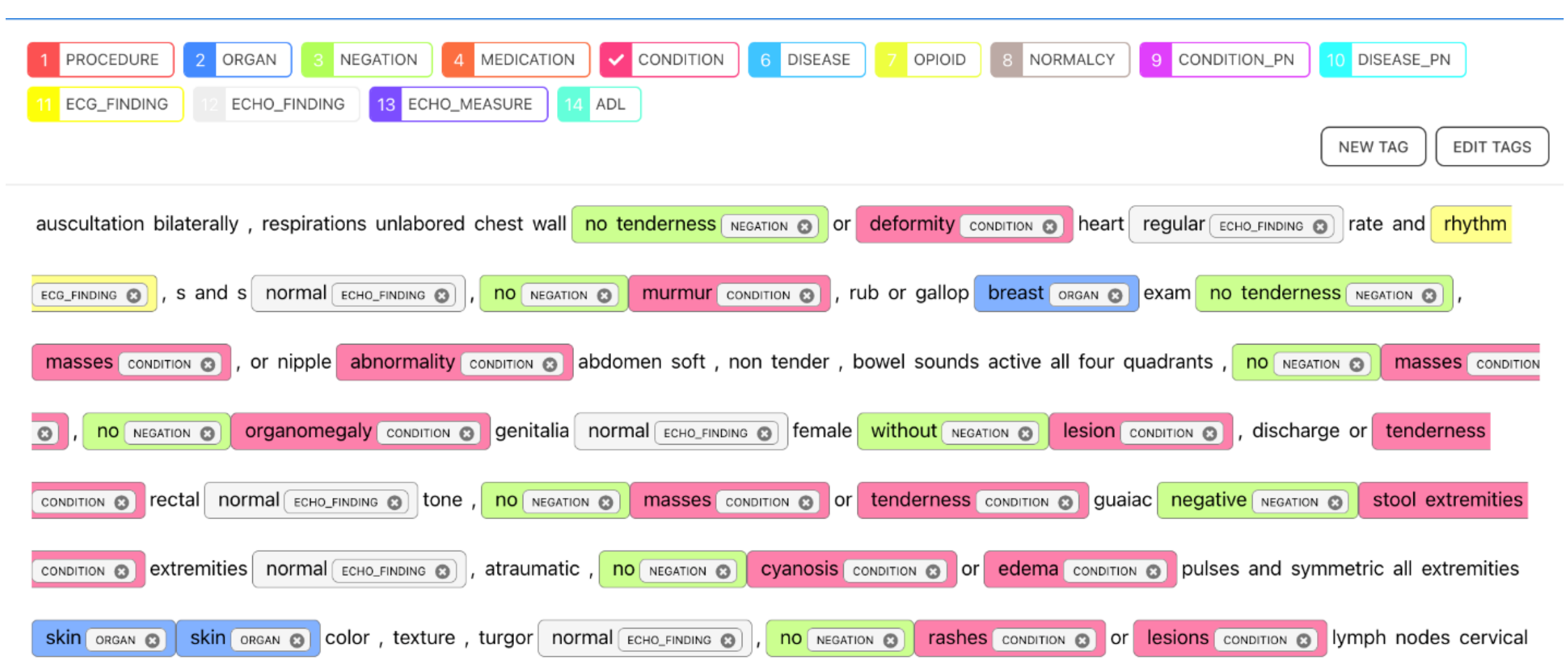

eFigure5. NER for Echocardiogram and ECG Data

Figure 1 : ECHO and ECHO parameters using NLP NER technique

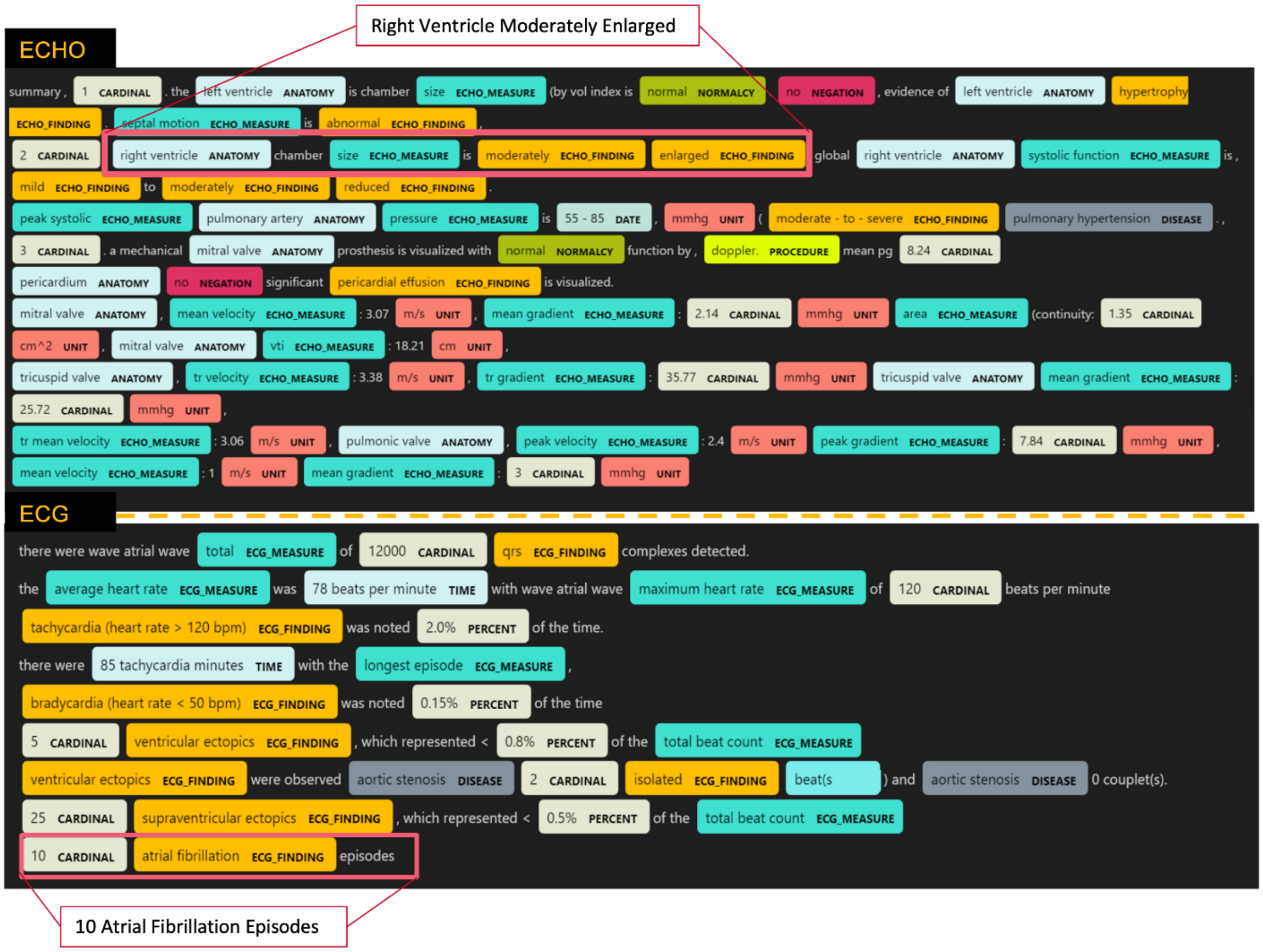

eFigure 6: Topic Modeling

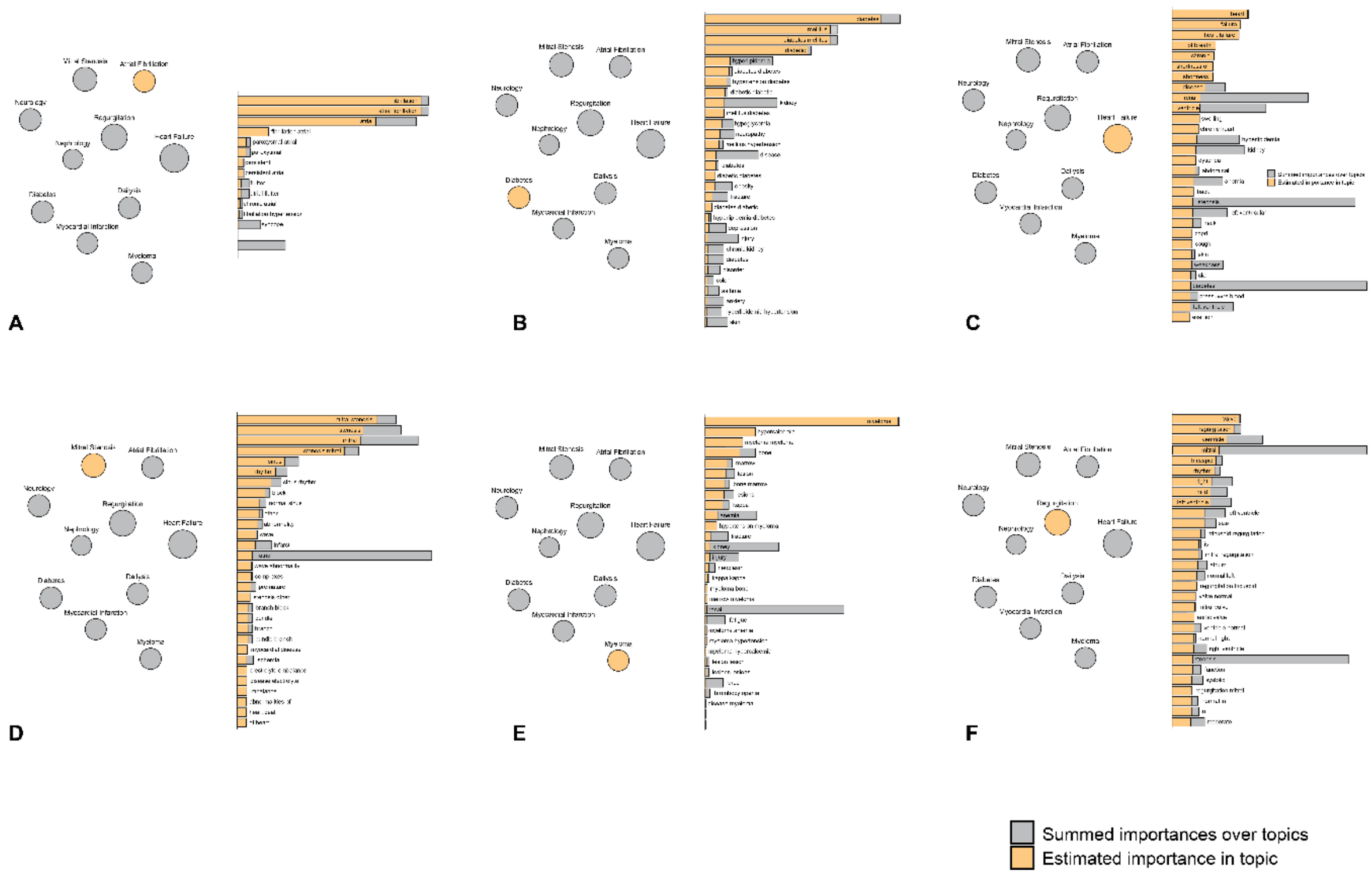

eTable 1. Deep Learning Algorithm Summary

Step	Details
**Step 1: Input Data**	Unstructured clinical notes processed using the Clinical ModernBERT tokenizer with a maximum sequence length of 2,048 tokens.
**Step 2: Transformer Embedding**	Clinical ModernBERT generates contextual token-level embeddings from clinical text.
**Step 3: Pooling**	Masked mean pooling applied to token embeddings to produce a fixed-length 768-dimensional representation per note.
**Step 4: Classifier (MLP)**	Multilayer perceptron with dense layers (256 → 768 units), ReLU activation, and dropout (0.2).
**Step 5: Output Layer**	Sigmoid activation layer for binary classification of ATTR amyloidosis presence.
**Step 6: Loss Function**	Binary cross-entropy loss optimized using the Adam optimizer.
**Step 7: Evaluation Metrics**	AUROC, precision (PPV), recall (sensitivity), and F1-score for imbalanced classification evaluation.
**Step 8: Training Strategy**	Early stopping based on validation loss with a patience of 5 epochs.
**Step 9: Class Imbalance Handling**	Inverse-frequency class weighting applied during training to address low disease prevalence.

eTable 2: Demographic Characteristics with Corresponding Classification Metrics for Cohort (ATTR- CM)

Characteristic	PPV	Sensitivity	Specificity	F1	AUROC
**Age Group (Years)**					
45-65	0.149	0.889	0.963	0.255	0.926
65-85	0.172	0.85	0.962	0.286	0.906
>85	0.152	0.925	0.96	0.261	0.942
**Race**					
Asian	0.125	1	0.97	0.222	0.985
Black or African American	0.241	0.852	0.96	0.375	0.906
White	0.142	0.889	0.962	0.245	0.926
**Gender**					
Female	0.135	0.895	0.96	0.234	0.927
Male	0.18	0.846	0.963	0.297	0.905
**Ethnicity**					
Hispanic or Latino	0.087	0.643	0.963	0.154	0.803
Not Hispanic or Latino	0.167	0.887	0.962	0.281	0.924
**Comorbidities**					
Hypertension	0.204	0.831	0.961	0.327	0.896
Chronic Kidney Disease	0.227	0.868	0.956	0.359	0.912
Diabetes Mellitus	0.211	0.864	0.961	0.339	0.913
Atrial Fibrillation	0.246	0.884	0.962	0.385	0.923

eTable 3: Named Entity Recognition Model Performance Metrics

Entity Type	Precision	Recall	F1 Score
DISEASE	0.994	0.998	0.996
CONDITION	0.989	1.000	0.994
PROCEDURE	1.000	1.000	1.000
ANATOMY	0.999	0.998	0.999
ECHO_MEASURE	0.996	0.990	0.993
ECHO_FINDING	0.989	0.997	0.993
NEGATION	1.000	1.000	1.000
NORMALCY	1.000	1.000	1.000
UNIT	0.999	0.999	0.999
CARDINAL	0.943	0.951	0.947
QUANTITY	0.954	0.805	0.873
PERCENT	0.970	0.953	0.961

eTable4. Comparator models overview

Model	Full name	Imbalance handling	Threshold selection	CI method
LOG	Logistic Regression	Inverse-frequency class weights	Max Youden J statistic	Bootstrap (n=1000)
BRFC	Balanced Random Forest Classifier	Internal (bootstrap balanced subsets)	Max Youden J statistic	Bootstrap (n=1000)
BBC	Balanced Bagging Classifier	Internal (balanced bootstrap samples)	Max Youden J statistic	Bootstrap (n=1000)
RUS	Random Under-Sampling Boosting	Internal (random under-sampling)	Max Youden J statistic	Bootstrap (n=1000)
XGB	Extreme Gradient Boosting	Inverse-frequency class weights	Max Youden J statistic	Bootstrap (n=1000)

eTable5. Hyperparameter tuning details

All hyperparameters were selected by a 4-fold stratified cross-validation grid search on the training set, optimizing AUROC.

**Model**	**Hyperparameter**	**Search space**	**Optimal value**	**Tuning method**
LOG	C (regularisation strength)	0.001, 0.01, 0.1, 1, 10, 100	1	4-fold CV grid search
LOG	penalty	l1, l2	l2	4-fold CV grid search
LOG	solver	lbfgs, saga	lbfgs	4-fold CV grid search
BRFC	n_estimators	100, 200, 500	200	4-fold CV grid search
BRFC	max_depth	None, 10, 20, 30	None	4-fold CV grid search
BRFC	min_samples_leaf	1, 2, 4	1	4-fold CV grid search
BRFC	max_features	sqrt, log2	sqrt	4-fold CV grid search
BBC	n_estimators	50, 100, 200	100	4-fold CV grid search

BBC	max_samples	0.5, 0.75, 1.0	1.0	4-fold CV grid search
BBC	base estimator max_depth	3, 5, None	5	4-fold CV grid search
RUS	n_estimators	50, 100, 200	100	4-fold CV grid search
RUS	learning_rate	0.1, 0.5, 1.0	1.0	4-fold CV grid search
RUS	sampling_strategy	auto, 0.5, 0.75	auto	4-fold CV grid search
XGB	n_estimators	100, 300, 500	300	4-fold CV grid search
XGB	max_depth	3, 5, 7	5	4-fold CV grid search
XGB	learning_rate	0.01, 0.05, 0.1	0.05	4-fold CV grid search
XGB	subsample	0.6, 0.8, 1.0	0.8	4-fold CV grid search
XGB	colsample_bytree	0.6, 0.8, 1.0	0.8	4-fold CV grid search
XGB	scale_pos_weight (imbalance)	Computed as N_neg / N_pos	~29 (CM), ~9 (MP)	Fixed

eTable6. Training and Evaluation settings

Setting	Description
Feature input	Patient-level Clinical ModernBERT embeddings (768-dimensional masked mean-pooled vectors from entity-summarised notes)
Outcome	Presence of ATTR amyloidosis ICD-10-CM code (E85.*) at patient level, excluding AL light-chain amyloidosis (E85.81)
Train / test split	80% training, 20% held-out test; stratified by outcome
Cross-validation	4-fold stratified CV on training set for hyperparameter tuning
Threshold selection	Maximum Youden J statistic (sensitivity + specificity − 1) on the CV validation fold
Confidence intervals	Bootstrap resampling, n = 1000, on the held-out test set
Evaluation metrics	AUROC, PPV, NPV, sensitivity, specificity, F1, best J statistic
Implementation	Python; scikit-learn (LOG, BRFC, BBC, RUS); XGBoost; imbalanced-learn

Abbreviations: LOG, logistic regression; BRFC, balanced random forest classifier; BBC, balanced bagging classifier; RUS, random under-sampling boosting classifier; XGB, extreme gradient boosting; CV, cross-validation; AUROC, area under the receiver-operating characteristic curve; PPV, positive predictive value; NPV, negative predictive value; CM, cardiomyopathy cohort; MP, mixed phenotype cohort.

ATTR-Mixed Phenotype Supplement

INSIGHT-MP Model: Explainable Artificial Intelligence to Identify ATTR-Mixed Phenotype (MP)

METHODS

Baylor Scott & White Health (BSWH) is a large integrated healthcare system in Texas with a diverse patient population and 13 million patient encounters yearly. The study sample comprised patients aged ≥18 years who had at least 1 BSWH health care encounter in EHR between April 1, 2020, and March 31, 2023, and an echocardiogram within one year before or after the index date of a qualifying diagnosis. Qualifying diagnoses, based on International Statistical Classification of Diseases, Tenth Revision, Clinical Modification (ICD-10-CM) codes, included HF (I50), ATTR amyloidosis (E85), and peripheral or autonomic neuropathy (G54 to G64, G90, G99). The cohort for development of the INSIGHT-MP model represented patients with ATTR-MP among HF patients with concomitant polyneuropathy and cardiomyopathy (**eFigure 1**).

Data Source, Study Outcomes, and the Annotation process were as described for INSIGHT-CM. Model Development likewise proceeded for INSIGHT-MP as for INSIGHT-CM, but in the cohort of patients with concomitant polyneuropathy and cardiomyopathy. INSIGHT-CM and INSIGHT-MP shared the same feature extraction and classification methods but targeted different datasets and populations. The INSIGHT-MP model used Balanced Random Forest Classifier (BRFC) to identify ATTR-MP cases among patients with HF who had neuropathy and cardiomyopathy.

Interpretability Analysis was conducted as for INSIGTH-CM, using Local Interpretable Model-Agnostic Explanations (LIME).

Statistical Analysis

Statistical analyses were conducted as for INSIGHT-CM.

RESULTS

Study Cohort

A total of 13,500 patients with HF who had neuropathy and cardiomyopathy and ≥1 encounters were identified from the BSWH EHR between April 1, 2020, and March 31, 2023. The mean age was 74.1±13.31 and 6,999 (51.8%) were female. Among these patients, 412 had ATTR-MP diagnoses. **Table 1-MP** shows the patient characteristics for this cohort.

Model Evaluation

To predict ATTR-MP (n=412) among patients with HF who had concomitant neuropathy and cardiomyopathy (n=13,500), the INSIGHT-MP achieved a sensitivity of 90.0% (95%CI 84.5-91.0), specificity of 86.1% (95%CI 82.5-88.5), PPV of 14.2% (95%CI 10.5-16.0), NPV of 99.7% (95%CI 99.6-99.7), and an F1 score of 24.5% (95%CI 18.5-27.0) (**Table 2-MP**). **Figure 1-MP** summarizes the performance of the best model and illustrates the classification summary panel, featuring AUROC and precision-recall curves with threshold adjustment, showcasing the model's adaptability across clinical thresholds. The classification summary performance for additional machine learning models, including metrics such as accuracy, precision, recall, F1-score, NPV, specificity, and AUROC is presented in **Table 2-MP** for the ATTR-MP cohort.

Table 3-MP highlights the metrics stratified by demographic characteristics for the ATTR-MP cohort.

Model Interpretability

LIME-based interpretability analysis revealed key features that machine learning models leveraged for identification of ATTR-MP. The top predictors for predicting ATTR-MP were carpal tunnel, joint-swelling, AF, dyspnea, weight loss, tingling, heat and cold intolerance, syncope, paresthesia, and claudication (**Figure 2-MP**).

Sources of Funding: This work was funded by AstraZeneca and generously supported by a philanthropic gift from Ashley and Greg Arnold.

Disclosures: MSK reports fees from Bayer and Novartis; **RLG** is a consultant/advisor, and/or has received grants or speaking /lecture fees from Alnylam Pharmaceuticals Inc, AstraZeneca, Eli Lilly and Company, Pfizer, and Johnson & Johnson; **AM**: Research grants from Pfizer, Ionis, Attralus, Cytokinetics, and Janssen and consulting fees from BridgeBio, Pfizer, Ionis, Lexicon, Attralus, Cytokinetics, BMS, Alnylam, Haya, Edgewise, Alexion, Akros, Prothena, BioMarin, Lexeo, AstraZeneca, Rocket and Tenaya; **ELP** has grants from Astra Zeneca, Boehringer Ingelheim, CSL Vifor, and Owkin**; MP and JV** are employed by AstraZeneca with ownership in AZ stock; and **JB** is a consultant to Abbott, American Regent, Amgen, Applied Therapeutic, AskBio, Astellas, AstraZeneca, Bayer, Boehringer Ingelheim, Boston Scientific, Bristol Myers Squibb, Cardiac Dimension, Cardior, CSL Bearing, CVRx, Cytokinetics, Edwards, Element Science, Faraday, Foundry, Innolife, Impulse Dynamics, Imbria, Inventiva, Ionis, Lexicon, Lilly, LivaNova, Janssen, Medtronics, Merck, Occlutech, Owkin, Novartis, Novo Nordisk, Pfizer,

Pharmacosmos, Pharmain, Prolaio, Regeneron, Renibus, Roche, Salamandra, Sanofi, SC Pharma, Secretome, Sequana, SQ Innovation, Tenex, Tricog, Ultromics, Vifor, and Zoll**; all** remaining authors report no conflicts.

Table 1-MP: Baseline characteristics of included patients.

Characteristic	INSIGHT-MP Cohort
**Age (years), mean (SD)**	74.1 (13.3)
**Body mass index mean (SD)**	31.1 (6.8)
**Sex**	
Male, n (%)	6501(48.2)
Female, n (%)	6,999 (51.8)
Unknown, n (%)	0 (0.0)
**Race, n (%)**	
American Indian or Alaska Native	67 (0.5)
Asian	121 (0.9)
Black	2,228 (16.5)
Native Hawaiian or Other Pacific Islander	27 (0.2)
White	10,854 (80.4)
Unknown	203 (1.5)
**Ethnicity**	
Hispanic	1,175 (8.7)
Non-Hispanic	12,244 (90.7)
Unknown	81 (0.6)
**Marital Status n (%)**	
Married	6,495 (48.1)
Single	2,137 (15.8)
**Smoking Status n (%)**	
Current	1,425 (10.6)
Former	5,715 (42.3)
**Deceased Status n (%)**	
Alive	10,193 (75.5)
Deceased	3,307(24.5)
**Prior Comorbidities n (%)**	
Hypertension	12,343 (91.4)
Chronic kidney disease	7,326 (54.3)
Diabetes mellitus	7,154 (52.9)
Atherosclerotic Cardiovascular Disease	7,697 (57.0)
Atrial fibrillation	6,257 (46.4)
Myocardial Infarction	4,070(30.1)
Cardiomyopathy	3,869 (28.7)
**Heart failure by LVEF n (%)**	

HFpEF (LVEF ≥50%)	6,354 (47.1)
Unknown	4,388 (32.5)
HFrEF (LVEF ≤40%)	1,574 (11.7)
HFimpEF (previous LVEF ≤40%; follow-up LVEF >40%)	638 (4.7)
HFmrEF (LVEF 41%–49%)	546 (4.0)
**Amyloidosis Diagnosis, n (%)**	412 (3)

LVEF: Left ventricular ejection fraction; HFpEF: heart failure with preserved ejection fraction; HFrEF: heart failure with reduced ejection fraction; HFmrEF: heart failure with mildly reduced ejection fraction; HFimpEF: heart failure with improved ejection fraction

Table 2-MP: Performance metrics of machine learning and deep learning models.

Model Name	PPV (%) (95% CI)	NPV (%) (95% CI)	Sensitivity (%) (95% CI)	Specificity (%) (95% CI)	F1 (%) (95% CI)	AUROC (%) (95% CI)	Screened Positive (%)
BRFC	14.2 (10.5-16.0)	99.7 (99.6-99.7)	90.0 (87.0-93.1)	86.1 (85.6-88.8)	24.5 (23.9-27.1)	93.6 (91.9-95.1)	16.2 (15.0 – 17.8)
XGB	14.4 (13.1-16.3)	99.8 (99.7-99.8)	91.1 (90.5-93.4)	86.1 (85.5-88.4)	24.8 (23.2-26.3)	95.7 (94.0-97.0)	16.3 (15.2 – 17.7)
BBC	14.2 (11.6-15.7)	99.5 (99.5-99.6)	75.5 (73.2-76.2)	79.2 (77.7-80.9)	23.9 (21.6-24.9)	90.5 (89.9-92.9)	22.5 (21.3 – 23.8)
LOG	11.8 (10.6-13.8)	99.5 (99.5-99.6)	84.4 (83.2-86.2)	83.9 (82.1-85.2)	20.7 (19.1-22.2)	91.4 (90.8-93.8)	18.2 (17.0 – 19.5)
RUS	8.8 (7.3-10.4)	99.2 (99.1-99.2)	72.3 (71.4-76.5)	80.7 (80.2-83.3)	15.7 (13.5-16.5)	85.7 (83.2-89.8)	21.5 (19.8 – 22.8)

LOG, Logistic Regression; BRFC, Balanced Random Forest Classifier; BBC, Balanced Bagging Classifier; RUS, Random Under Sampling and Boosting Classifier; XGB, Extreme Gradient Boosting Classifier; NPV, negative predictive value; PPV, positive predictive value.

Table 3-MP: Demographic Characteristics with Corresponding Classification Metrics for the INSIGHT-MP Cohort

Characteristic	PPV	Sensitivity	Specificity	F1	AUCROC
**Age Group (Years)**					
45-65	0.108	0.923	0.857	0.194	0.89
65-85	0.158	0.902	0.874	0.269	0.888
>85	0.078	1	0.85	0.145	0.925
**Race**					
Asian	0.333	1	0.882	0.5	0.941
Black or African American	0.196	0.952	0.852	0.325	0.902
White	0.109	0.902	0.869	0.195	0.885
**Gender**					
Female	0.092	0.96	0.868	0.168	0.914
Male	0.158	0.898	0.865	0.268	0.881
**Ethnicity**					
Hispanic Or Latino	0.049	1	0.881	0.093	0.941
Not Hispanic or Latino	0.133	0.917	0.865	0.232	0.891
**Comorbidities**					
Hypertension	0.143	0.914	0.864	0.247	0.889
Chronic Kidney Disease	0.167	0.932	0.861	0.284	0.897

Diabetes Mellitus	0.143	0.977	0.867	0.25	0.922
Atrial Fibrillation	0.151	0.932	0.858	0.259	0.895

Figures

Figure 1-MP: : Performance metrics of machine learning models for ATTR-MP (A) Classification Summary ATTR-MP (B) Confusion Matrix ATTR-MP (C) AUROC Curve ATTR-MP (D) Precision – Recall Curve ATTR-MP

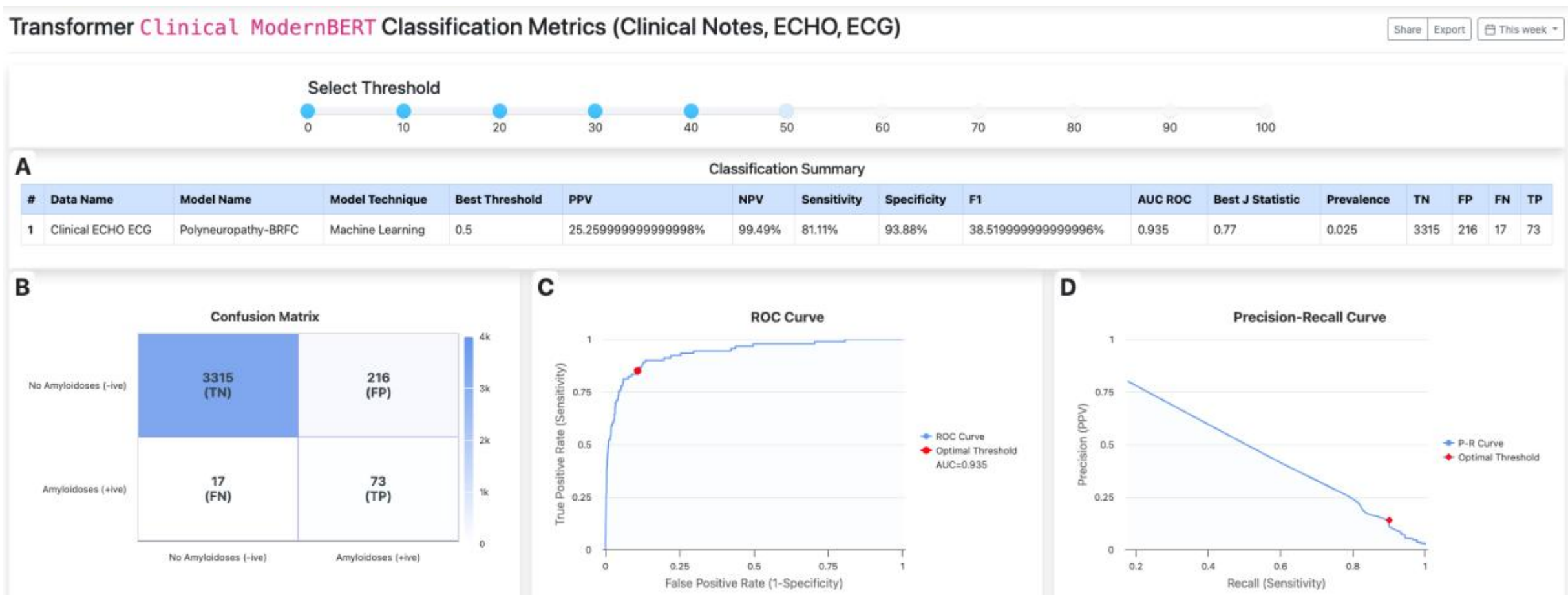

Figure 2-MP: Top predictors by Local Interpretable Model-Agnostic Explanations for ATTR-MP. (A) Lime Model Explanations ATTR-MP (B) Named Entity Recognition (NER) ATTR-MP

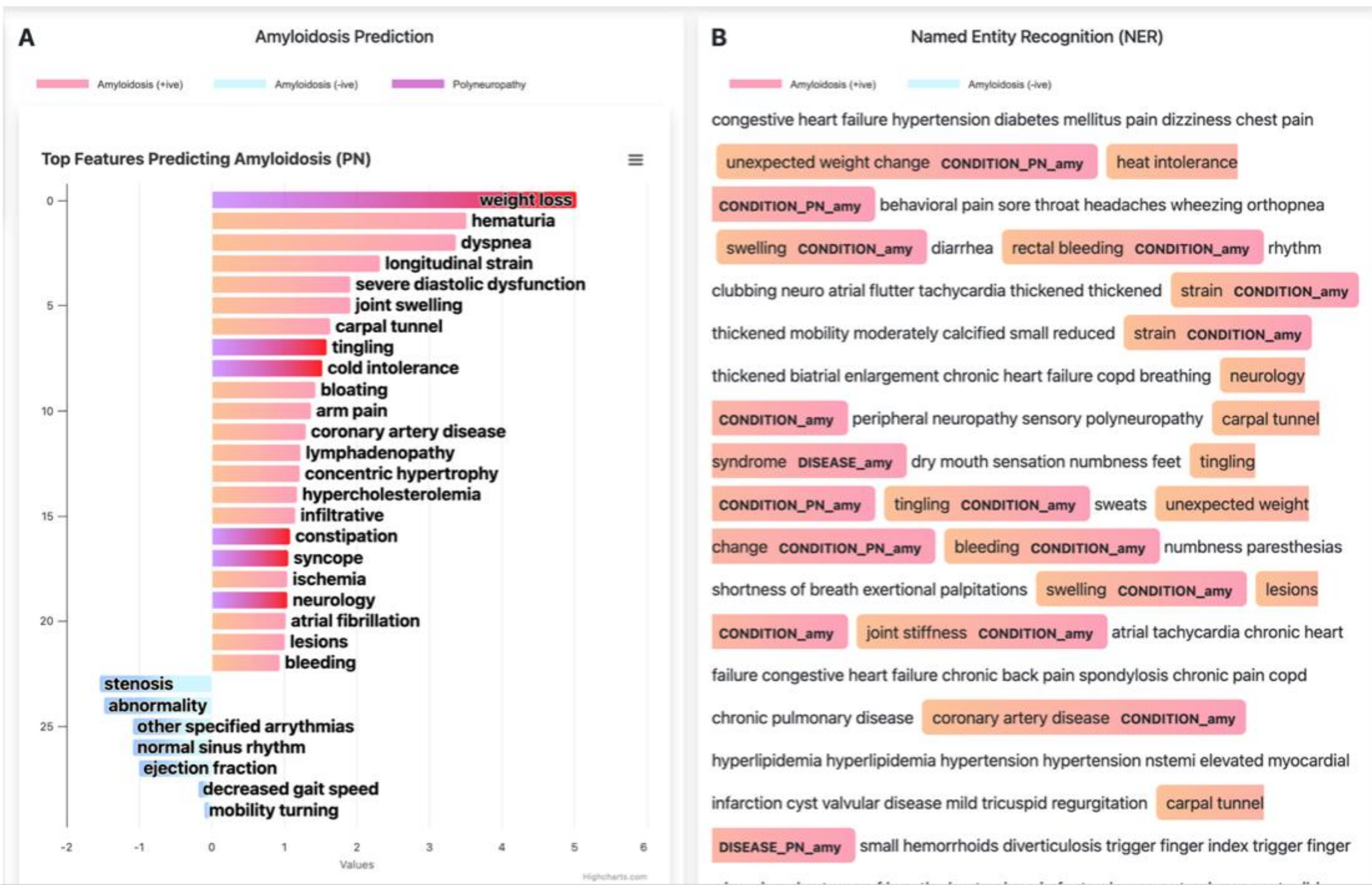

Supplement: Supplementary file 1 [file mmc1.pdf]
